# Investigation of the Fabrication Suitability, Structural Performance, and Sustainability of Natural Fibers in Coreless Filament Winding

**DOI:** 10.3390/ma15093260

**Published:** 2022-05-01

**Authors:** Pascal Mindermann, Marta Gil Pérez, Jan Knippers, Götz T. Gresser

**Affiliations:** 1 Institute for Textile and Fiber Technologies, University of Stuttgart, Pfaffenwaldring 9, 70569 Stuttgart, Germany; 2 Institute of Building Structures and Structural Design, University of Stuttgart, Keplerstraße 11, 70174 Stuttgart, Germany; m.gil-perez@itke.uni-stuttgart.de (M.G.P.); jan.knippers@itke.uni-stuttgart.de (J.K.); 3 German Institutes of Textile and Fiber Research Denkendorf, Körschtalstraße 26, 73770 Denkendorf, Germany

**Keywords:** coreless filament winding, natural fibers, bio-based resin, four-point bending testing, life-cycle assessment, embodied energy, global warming potential

## Abstract

Coreless filament winding is an emerging fabrication technology in the field of building construction with the potential to significantly decrease construction material consumption, while being fully automatable. Therefore, this technology could offer a solution to the increasing worldwide demand for building floor space in the next decades by optimizing and reducing the material usage. Current research focuses mainly on the design and engineering aspects while using carbon and glass fibers with epoxy resin; however, in order to move towards more sustainable structures, other fiber and resin material systems should also be assessed. This study integrates a selection of potential alternative fibers into the coreless filament winding process by adapting the fabrication equipment and process. A bio-based epoxy resin was introduced and compared to a conventional petroleum-based one. Generic coreless wound components were created for evaluating the fabrication suitability of selected alternative fibers. Four-point bending tests were performed for assessing the structural performance in relation to the sustainability of twelve alternative fibers and two resins. In this study, embodied energy and global warming potential from the literature were used as life-cycle assessment indexes to compare the material systems. Among the investigated fibers, flax showed the highest potential while bio-based resins are advisable at low fiber volume ratios.

## 1. Introduction

Fiber-reinforced composite materials are widely used in aerospace, automotive, energy, and marine applications because of their favorable material properties, such as high mass-specific stiffness and strength [1,2,3], corrosion and chemical resistance [4], electrical and thermal isolation, low thermal expansion [5] as well as design flexibility. Depending on the target component geometry, different manufacturing processes [6] can be selected, such as braiding, pultrusion, filament winding, tape laying, compression molding, or vacuum infusion.

Coreless filament winding (CFW) emerged in the field of construction, enabling an automated [7] and FE-supported design process [8] for large-scale [9] building components, which can be individualized. In CFW, rovings are placed individually around spatially arranged anchors [10], held in place by a winding fixture. The fibers are impregnated with a thermosetting resin before or during the winding session and the winding session includes the execution of the winding plan. The component shape is not defined by a mold surface [11] but by the interaction of the fibers and the sequence in which the roving connects the anchors, called the winding syntax. Once the component is wound, it needs to be cured in an oven to achieve its final mechanical properties. CFW requires an integrative design, engineering, and fabrication approach which relies on the interaction of physical prototyping, testing, and simulation due to the lack of building codes [12].

Carbon and glass fiber-reinforced plastics (C/GFRPs) with epoxy resins have been utilized almost exclusively in CFW. The design language of CFW is well suited for intricate framework structures, matching the property profile of such high-performance materials, but it is also optimal for lattices [13,14] and shells [15] in lightweight structural applications. Examples are found in recent technology demonstrators [12,16] and research trends, which are centered around integrated structural design and technical advancements [17,18,19] in fabrication and digital design.

The research efforts to promote CFW in the construction sector are motivated by the increasing worldwide demand for building floor space in the following decades [20]. CFW allows the efficient fabrication of building systems as it was proved with the application of the technique into a multi-story installation [16]. Current state-of-the-art construction methods are not sufficiently digitized or automated to achieve a higher level of productivity in the building sector, while steel/concrete-based material systems are not sustainable enough to meet the needs of resource efficiency and significant reductions in CO_2_ emissions. Filament winding is one of the less energy-intensive manufacturing processes compared to other composite fabrication techniques [21]; however, the focus of CFW to C/GFRPs and petroleum-based epoxy resins must be reconsidered, and other alternative options must be assessed to effectively fulfill this objective.

**Figure 1:**
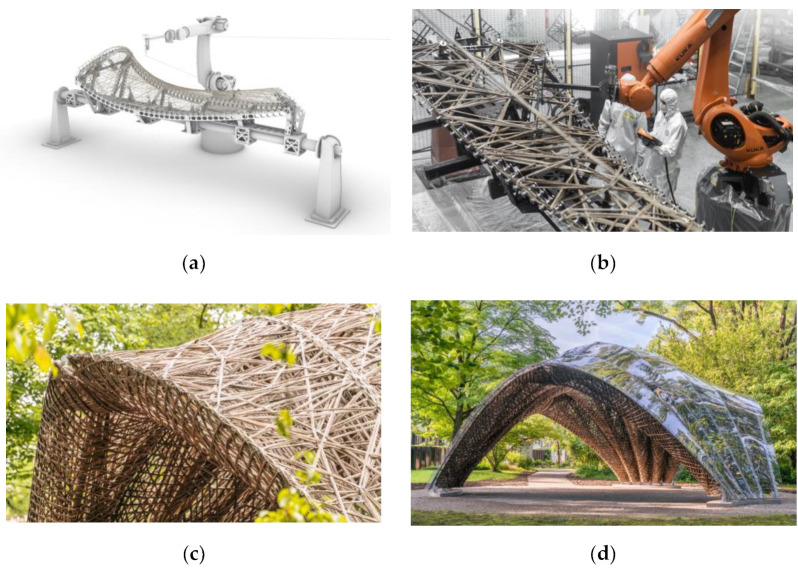
First large-scale application of natural fibers in a coreless wound structure, the LivMatS pavilion [24]. (**a**) Digital planning of the winding setup and process; (**b**) state-of-the-art multi-axis robotic fabrication setup using a stationary resin bath impregnation method and an external creel; (**c**) natural fiber component (flax and sisal mix) during building erection; (**d**) final pavilion. © ICD/ITKE/IntCDC University of Stuttgart.

There are several alternative fiber materials to carbon and E-glass, including both technical and natural fibers. The usage of natural fibers in the general field of fiber-reinforced plastic manufacturing is increasing to meet the growing industrial demand for more sustainable and structural components. This change is motivated by customer awareness, increasingly including other non-technical or economical parameters, such as ecological or social issues [22]. For example, the German automotive industry consumes 19 kt of natural fiber composites annually, with 64% flax, 11% jute/kenaf, 10% hemp, and 7% sisal [23]. While several textile processes were already compatible with natural fibers, others needed adaptations to handle the material characteristics of natural fiber products. Although the initial deployment of natural fibers [24] (Figure 1) and bio-based resins [25] have shown the potential and the suitability of these alternative materials to CFW, these applications have also proven that a more extensive assessment in terms of fabrication, structural behavior and sustainability is needed.

In terms of fabrication, the rovings must endure the handling characterized by fast variations in fiber tension during the winding [25,26] and significant deflections [27] at the anchor elements. These challenges become exponential with materials, such as natural fibers, which present an insufficient strength when uncured, as the robot will break individual fibers or filaments. Therefore, adjustments in the fabrication process are crucial. The fabrication setups can be classified according to the used impregnation method and fiber source location. With a conventional stationary resin bath [28] on the ground, large quantities of rovings can be impregnated, since no installation space restrictions apply. In order to avoid dripping rovings traveling through the fabrication setup, different impregnation methods were developed in recent years that integrate the impregnation cartridges [25,29] in the winding head. The challenge here is sealing the impregnation chambers without damaging the rovings or restricting the winding head orientation. In those systems, either a resin reservoir can be accommodated in the winding head, or the resin can be fed through a supply line. There are several options for positioning the fiber source: an external stationary creel offers the highest capacity but introduces free-spanning rovings/fiber bundles into the system, while a creel mounted on the robot system limits fiber traveling to the area between the robot base and winding head, but it has a lower capacity. Individual fiber spools integrated into the winding head have the lowest capacity, but completely eliminate fiber traveling. Which fabrication setup configuration should be selected also depends strongly on the characteristics of the deployed fiber and resin materials.

Coupled with the fabrication challenges, the structural engineering of these systems is also unconventional. The geometrical uncertainties and material deviations require the development of structural design methods integrated during the computational design process in a continuous feedback loop [12,16]. The production process (e.g., harvesting or fiber extraction) of natural fibers produces significant variability in the mechanical properties [30], and they can also present a weak fiber-matrix interface [31], directly linked to low reproducibility. These additional issues make it necessary to integrate the material characterization into the design workflow [24].

The potential of these new material systems should be analyzed considering their mechanical performance along with their sustainability aspects. For this assessment, the specific amount of material needed to fulfill a specific strength of stiffness requirement can be compared to sustainability indexes [32]. State-of-the-art life cycle assessment (LCA) can be deployed to holistically evaluate the different materials’ sustainability while a complete LCA assesses the environmental aspects and potential impacts of the entire product’s life cycle [33]; however, previous LCA studies have shown that natural fiber composites are superior during the use and end-of-life periods, making the production phase the actual key to the final environmental impact [32].

Considering that only a limited number of alternative materials have been implemented in CFW in previous studies [24,25], the objective of the presented research was to preselect and investigate a larger variety of alternative materials and derive adaptations of the fabrication process and equipment. Then, the structural performance was assessed concerning the environmental impact of each material. For this study, only two sustainability markers were used: the embodied energy and the global warming potential (GWP) for the production of the selected fiber and resin products. Along with this experiment-based revision of several alternative winding materials, the processing suitability was also investigated. The final aim of this study was to investigate which option is superior: the efficient use of high-performance lightweight materials or the deployment of low-performance materials which are more sustainable but require a higher material consumption.

## 2. Materials and Methods

### 2.1. Composite Winding Material Selection

In a first step, a collection of material properties for potential fiber and matrix candidates was extracted from an exhaustive literature review (Table 1/Table A1). This review aimed to cover the range of properties investigated or tested in previous studies to provide an overview of the current research state of alternative fiber materials. Especially in the case of natural fibers, the manufacturers, suppliers, and testing conditions significantly influence the scattering of the data [23], making any general investigation harder. The matrix system selection was limited to thermoset resin since others would require extensive adjustments of the CFW process and equipment, which would be beyond the scope of this study.

There were several criteria for the pre-selection of fiber materials. First, many plant-based fibers were excluded since they are not locally produced in Europe, such as bamboo, banana, and pineapple, keeping logistics minimal to contribute to the sustainability. Another important aspect was that the materials should be producible in quantities relevant for the construction. Further criteria were set based on the mechanical performance parameters. Materials with excessively high elongation at break are unsuitable for CFW, as they cannot withstand the constant fiber tension during winding. This issue affects spider silk, polyester, and palm fiber. Moreover, since stiffness and strength are required simultaneously for structural application, materials with one significantly low value were excluded, as with the case of cotton and coir. Another parameter would be the price per kilogram, which excludes boron fibers. Furthermore, the ratio between the mechanical performance parameters and the sustainability parameters should be in a range that could be superior to other fiber materials. Wool, for example, has a relatively low strength compared to its GWP and loses approx. 20% of its capacity when wet [34]. In addition, the ratio of mechanical performance and density is essential to realize lightweight structures, excluding other fibers such as sisal. In the last step, some materials with interesting properties were included, such as stainless steel, which can be melted down at the end of life, or hemp, which was available as a card sliver, reducing the energy needed for its production.

A conventional epoxy resin was compared to a bio-based epoxy resin. Investigating other types of thermosetting resins was out of the scope of this study for several reasons. Polyester resins are less performative than epoxy resins [35], especially regarding strength. Resin products containing solvents, such as styrene, which are classified as toxic [36] and probably carcinogenic [37], cannot be used in large-scale CFW applications due to the lack of ventilation in the setup space. Polyurethane resins show relatively short pot life, while phenolic resins degrade under persistent ultraviolet exposure, and polyimide resins exhibit a noticeable water absorption [38] and are expensive. These properties would not be preferable for coreless wound structural components in construction applications. For these resin types, bio-based substitutes are available [39].

**Figure 2:**
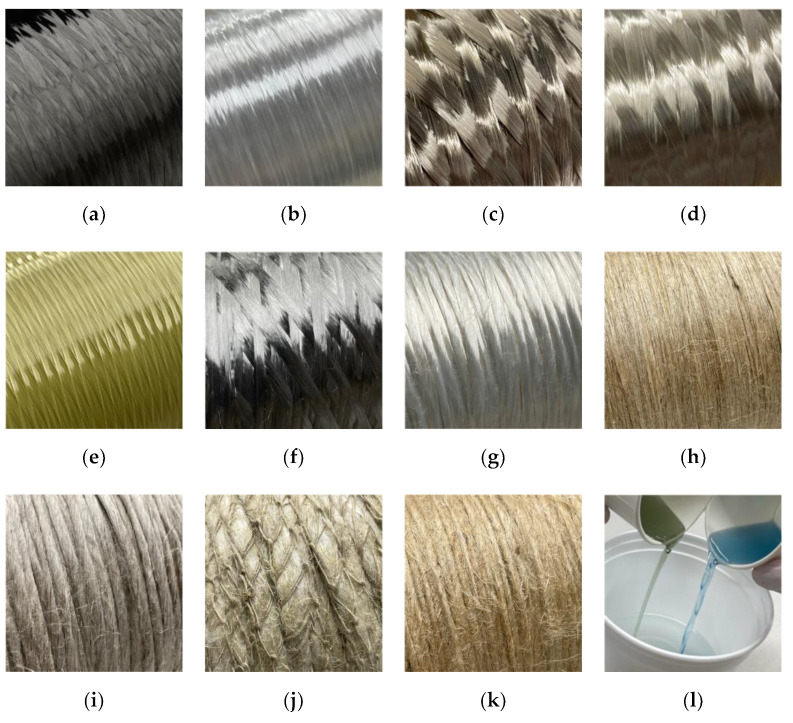
Material selection for this study: (**a**) carbon; (**b**) glass; (**c**) basalt type 1; (**d**) basalt type 2; (**e**) aramid; (**f**) stainless steel; (**g**) viscose; (**h**) flax tape; (**i**) flax yarn; (**j**) hemp (crocheted card sliver); (**k**) jute; (**l**) epoxy resin.

Based on this screening, the following materials (Figure 2) were selected for this study: carbon (C), E-glass (G1), S-glass (G2), two types of basalt (B1, B2), aramid (A), stainless steel (S), viscose (V), flax as tape (F1) and yarn (F2), hemp (H), and jute (J). All samples were fabricated using a bio-based resin, while for the carbon (C*) and the flax tape (F1*), another set was produced with conventional petroleum-based resin for comparative reasons.

#### 2.1.1. Fiber Materials

Carbon and glass acted as a benchmark to evaluate the alternative fibers since the CFW process was developed primarily based on these two materials. All the selected technical fiber products exhibit high consistency in their material parameters; however, plant-based fibers are natural products with significant variations in material parameters between batches and along the fiber bundle. These variations come from defects in the short individual fibers or the production of long yarns, as the fibers need to be held together by stapling them [23]. Viscose fiber does not exhibit such variations, as the continuous fibers are man-made in a wet spinning process from cellulose wood pulp.

In addition, the engineered fiber products come with a sizing that ensures good fiber-matrix adhesion to a particular matrix system. In the case of natural-based fiber products, the presence of hydroxyl groups gives a highly hydrophilic nature to the fiber, resulting in low moisture resistance and, consequently, in a weak interfacial fiber-matrix bonding [30]. Chemical treatments [40] can be performed on the yarn to increase adhesion. It should be noted that the fiber products used in this study did not receive any chemical post-treatment after their acquisition. Other aspects relevant in an architectural setting are flammability, fire resistance, and hygrothermal ageing when exposed to the environment [41]. All these effects can compromise the mechanical properties of the composite; for example, a 60% increase in relative humidity can reduce the elastic modulus of plant-based fibers to 35% [42].

The production of long fiber bundles is also not as easy as with technical fiber. Twisting is commonly used to avoid the breakage of fibers that are just stapled together, producing better friction between fibers [23]; however, the twist reduces the permeability of the fiber, resulting in worse fiber impregnation [43]. Moreover, it also produces a change in directionality that translates in misalignments with axial loading, reducing the composite tensile strength [43]. Nevertheless, all these challenges and detriments can be counterbalanced during the design and fabrication processes or by the deployment of chemical treatments before or after the winding.

Carbon fibers exhibit excellent processability using state-of-the-art CFW equipment. Its high mass-specific mechanical performance parameters, especially stiffness, are in accordance with the CFW design language, allowing the realization of filigree truss structures. It also incorporates a high chemical resistance. As a disadvantage, carbon fibers are obtained from non-renewable petroleum-based raw materials such as polyacrylonitrile (standard) or pitch (high-performance). It is also possible to produce carbon fibers from a renewable material by converting viscose fibers into carbon fibers, although the resulting fibers are less performative. For its production, the carbon concentration in the precursors is gradually increased in an energy-intensive stretching process. This process involves oxidative stabilization and carbonization/graphitization steps under an inert gas atmosphere, using high temperatures. Carbon fibers are relatively expensive and practically non-recyclable [44].

Glass fibers show good mechanical performance at a lower price level than carbon. It is more suitable for high-strength applications than for high-stiffness ones. The processability in the CFW process is slightly worse than that of carbon, but still suitable. Glass fibers are spun from molten silica sand and have high chemical resistance. Several types of glasses with different properties can be created depending on their chemical composition. This study includes E-glass with a market share of about 99% [35] as a benchmark and S-glass with a higher strength, temperature, and chemical resistance. Another interesting feature is that glass fiber composites can be translucent when combined with certain resin types. The disadvantages of glass are its high density, low fatigue resistance, and non-recyclability [44]. Additionally, single filaments break more easily when handling the material compared to carbon fibers.

Aramid fibers combine high mass-specific tensile strength at a lower density than glass with high impact resistance. Aramid is spun from petroleum-based raw materials dissolved in sulfuric acid and its production is a very energy-intensive process. The fiber exhibits a low thermal expansion, similar to carbon, but achieves only lesser adhesion to the resin. It absorbs moisture and is sensitive to ultraviolet radiation. Although it is flammable, it features a good-natured behavior in the case of fire.

Two basalt fiber products were selected for this study, made from raw material with different chemical compositions. The fibers are spun from a melt of crushed volcanic rock and their absolute stiffness and strength values are slightly higher than glass fibers. In high-temperature applications, the values fall below those of glass fibers [45].

The good mechanical performance of stainless steel (1.4404) fibers is, compared to other technical fibers, relativized by its very high density. As a metal, it offers the possibility of being fully recycled by melting down. The stainless-steel fibers’ high electrical and thermal conductivities are not the primary concern in structural applications; however, if combined with carbon fibers, their high ductility can positively influence the failure behavior of components while maintaining a similar stiffnesses.

For flax, two products were also selected, a circular yarn and a flat tape. The tape allows for having parallel fibers, whereas the yarn has 20 tpm (turns per meter). Among the plant-based fibers, flax has the highest stiffness. In contrast to carbon fibers, flax has a lower density since it presents a hollow fiber structure. The flax yarn used had a European origin and received only an alkali washing by the manufacturer While the bio-degradable flax fibers were extracted from the stem of their plant.

Hemp offers slightly lower mechanical properties than flax at a lower price. It was selected for the investigation as it comes as a crochet card sliver, which requires less energy for its production but can also pose challenges for the handling in the CFW process. By eliminating processing steps, such as spinning, energy and CO_2_ can be saved. Cotton was used as a crochet thread, to bind the individual fibers of the hemp together to a coherent fiber bundle. The density of the hemp card sliver used (0.86 g/cm^3^) was lower than the literature values for hemp yarns (1.4–1.5 g/cm^3^, Table 1). The individual hemp fibers were obtained from the stem of the hemp plant and were also bio-degradable.

Jute fibers have a lower mechanical performance than flax fibers and a lower density and price, making them an attractive option. The jute yarn used for this study presented a twist of 65 tpm. It also exhibited a pronounced hairiness, which brought challenges to the winding process. The jute fibers were extracted from the stem of their plant and were also bio-degradable.

As mentioned earlier, viscose fibers are regenerated fibers made from cellulose wood pulp. They are supplied not in the form of a twisted yarn but as endless parallel fibers in a roving with constant material parameters. Viscose fibers have a lower stiffness but higher strength and elongation at break values compared to other selected plant-based fibers. The fibers are spun from a chemically treated spinning solution. These synthetic fibers also offer biodegradability. The selected roving has a low twist of 25 tpm for protection.

The required parameters and properties of the selected fiber materials are collected in Table 2.

Based on the datasheet or literature values, the tear length calculated for the dry fiber material gave an initial overview of what ranking could be expected later in the structural testing. With almost 250 km, the carbon fibers were first in this selection, followed by aramid, S-glass and E-glass, and then basalt fibers at 118–70 km. The listed natural fibers range reached up to 82 km. The highest values within this range were found in the hemp and flax tape. Flax yarn showed lower values compared to the tape. Moreover, the jute exhibited the lowest tear length at 34 km. Stainless steel fibers performed worse than all listed materials due to its high density.

**Figure 3:**
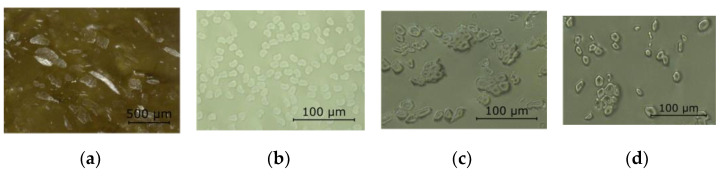
Microsections of some dry fiber bundles embedded in casting resin. (**a**) Hemp; (**b**) viscose; (**c**) flax tape; (**d**) flax yarn.

Using microsections (Figure 3) of dry fiber bundles embedded in casting resin, the mean fiber diameter was determined, as it was not reliably known for all selected fiber materials. The pentagonal contour of the flax fibers was observable in the tape and the yarn cases. The viscose fiber featured a kidney-shaped cross-section, whereas the hemp, as a card sliver, exhibited an irregular fiber contour.

#### 2.1.2. Matrix Materials

A bio-based epoxy resin recently introduced in CFW [25] was investigated in comparison to the petroleum-based epoxy resin commonly used in CFW demonstrators [48] (Table 3). The resin selection was narrowed to two materials to complement the extensive fiber materials study. Since variation in the fiber volume ratio can be expected between the different fiber materials, the impact of the resin on the component sustainability parameters must be taken into account. Including a bio-based resin in this study reduced the ecological impact of samples with a lower fiber volume ratio.

The selected bio-based resin system reduced the embodied energy by 70% and GWP by 68% compared to the conventional petroleum-based epoxy resin. This bio-based aliphatic epoxy resin system consisted of three components: epoxidized triglycerides as resin, polycarboxylic anhydride as a hardener, and an imidazole-based accelerator. The epoxidized triglycerides were synthesized entirely from a plant base. Its thermal curing should be above 120 °C for up to several hours, depending on the component thickness. It is also sensitive to humidity, therefore the fibers should be dried before winding. The selected petroleum-based resin cures at room temperature but requires tempering at 110 °C for 180 min.

### 2.2. Adjustments of the Winding Equipment and Process

**Figure 4:**
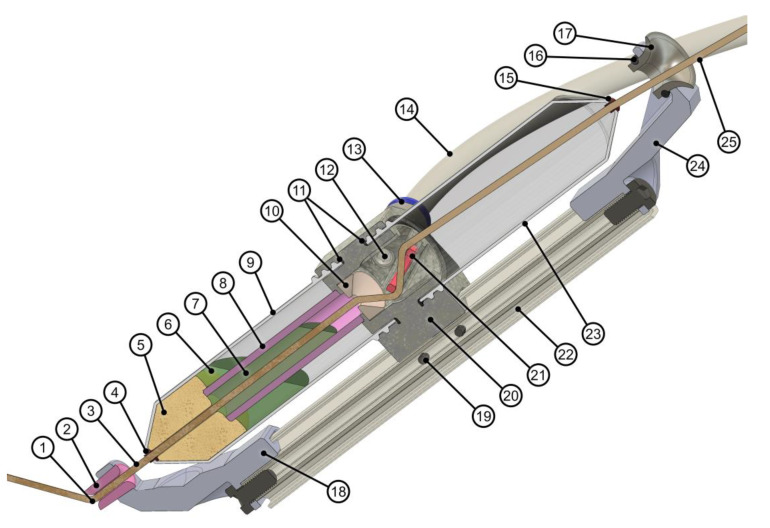
Sectional view of the developed winding head in CAD. 1: tool center point, 2: conical ceramic nozzle, 3: impregnated roving/fiber bundle, 4: ceramic eyelet (inside cartridge), 5: horizontally cut sponge, 6: outer resin chamber, 7: inner resin chamber, 8: ceramic tube, 9: lower impregnation cartridge, 10: ceramic spindle eyelet, 11: rubber gasket and silicon tape sealed threaded connection, 12: resin inflow, 13: resin supply tube connector, 14: resin supply tube, 15: ceramic eyelet (outside cartridge), 16: rubber ring, 17: chrome coated funnel, 18: nozzle holder, 19: cartridge holder fastening, 20: 3D-printed cartridge holder, 21: ceramic ring, 22: aluminum profile, 23: upper impregnation cartridge, 24: fiber intake holder, 25: dry roving/fiber bundle.

A winding head was developed to produce samples utilizing alternative materials and to investigate their impact on the winding equipment and process. Based on a previous design of a head-integrated impregnation system [25], the cartridge-based technique was further improved and adapted to the characteristics of the selected alternative fibers (Figure 4). Due to the theoretically unlimited pot life of the bio-based resin used, the resin supply hose required less frequent replacements.

The impregnation chamber consists of two polypropylene conical centrifuge tubes, which are diametral attached to a custom 3D-printed holder. All elements are attached to a 20 mm aluminum profile, which can be used as a handle or mounted to a robot. Due to the expected high fiber and resin consumption, it was decided to use an external material feed separately. A peristaltic pump supplies the impregnation chamber with already mixed resin and can realize a constant volume flow or, after one-time calibration, it can also dispense absolute volumes. The fibers were pulled off by the winding motion from an external creel with passive tension control. For drying the natural fibers in advance, a continuous dryer can be integrated between the creel and the winding head. As a second option, smaller fiber amounts can be dried discontinuously in an oven.

The fiber enters the winding head via a chrome-coated funnel with a large curvature to prevent fibers with even widely varying diameters from getting stuck when approaching at a shallow angle. Ceramic eyelets are placed in the drilled holes at the conical parts of the impregnation cartridges to cover the edges in the fiber traveling direction. Due to the different fiber bundle diameters of the material selection, eyelets with various diameters can be utilized. Tolerances should be included when selecting the eyelets because of the additional variation in diameter along the natural fiber bundle. The eyelets prevent damage to the passing fiber bundle and avoid protruding individual fibers from being cut off and accumulating as fragments. These problems are especially noticeable with more hairy fibers.

A higher fill level, eventually extending into the upper cartridge, improves impregnation due to more prolonged contact between the resin and fiber; however, it also increases leakage at the lower ceramic eyelet due to the higher resin pressure and it increasingly restricts the freedom of head orientations, which are possible without leakage at the upper ceramic eyelet. The 3D-printed threads for the cartridges are sealed by a rubber gasket and silicon sealing tape and shielded by an inner wall of the cartridge holder.

The holder is made by laser powder bed fusion from aluminum due to its higher chemical resistance compared to the formerly used polylactic acid. The resin supply tube connects laterally to avoid interference with the winding fixture, the component, and the dry fiber feed. The cartridges can be changed without disassembling the head.

**Figure 5:**
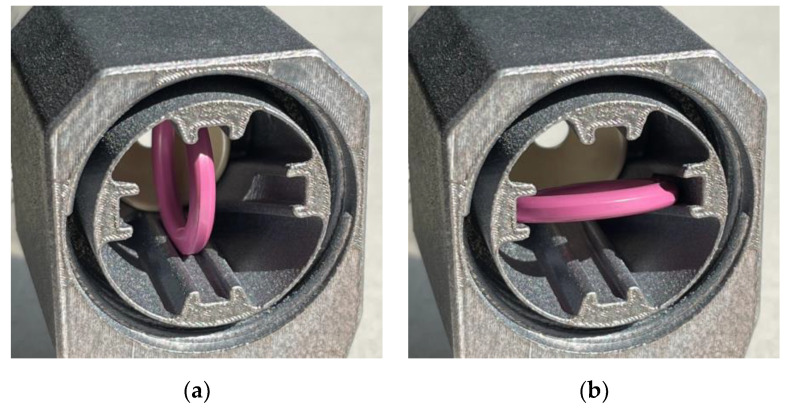
Install positions of ceramic ring in the cartridge holder in a rear view. (**a**) The fiber bundle passes the ring first on the left, then on the right, because of the resin inflow position; (**b**) the fiber bundle passes the ring first on the top, and then on the bottom, or the other way around depending on the required curvature.

Inside the chamber of the cartridge holder, a ceramic ring can be installed to improve impregnation. It can be positioned in two orientations to deflect the fiber bundle with three different intensities. The setting, shown in Figure 4, is the most intense deflection. Other options with less deflection include threading the fiber bundle below and above the ring or installing the ring vertically (Figure 5). The ring should be set to the lowest deflection with sufficient impregnation to avoid unnecessary fiber tension. The introduction of the ring enables more control over the impregnation than in previous systems [25,29]. It is the main contributor to friction-induced fiber tension. A lower fiber deflection decreases friction and fiber tension. Therefore, a further reduction of the fiber tension is only possible by adjusting the setting at the creel.

Then, a ceramic spindle eyelet centers the fiber bundle to reduce interaction with the sponge below. The fiber bundle enters the lower cartridge, separated by a ceramic tube into an inner and outer chamber. If the resin fill level falls below its recommended minimum value at the top edge of the cartridge holder, the lower cartridge separation facilitates that the contact time between the fiber and the resin remains as long as possible. The outer chamber provides a resin reservoir connected to the inner chamber by the resin-saturated sponge, namely, an open-cell polyurethane foam. The function of the sponge is to reduce leakages, helping with impregnation, and absorbing fiber fragments.

After leaving the impregnation chamber through another eyelet, the fiber bundle passes through a conical ceramic nozzle. Compared to the eyelets, it offers a larger fillet radius, prevents the transmission of lateral forces to the cartridges, exposes the tool center point (TCP) from the winding head, and allows damage-free collisions by swerving upwards. Sudden increases in the TCP velocity should be avoided to keep the fiber tension low, especially when processing natural fibers. The design of the winding head allows for fiber bundle retraction if the ceramic ring is not installed or does not become loose. The maximum retraction length is given by the distance from the upper ceramic eyelet to the current resin fill level in the system.

### 2.3. Structural Testing and Sample Parameters

The objective of the structural testing was to obtain comparative characteristic values for the mechanical performance parameters of the complete selection of materials and to provide a comparative evaluation of them for the subsequent sustainability assessment. In this context, the sample fabrication should exhibit the CFW process characteristics as comprehensively as possible. Due to the wide variation in bundle diameters of the selected fibers, it is not easy to design a setup that keeps the relevant cross-sectional area of the samples constant for all materials. In addition, due to the variability of the mechanical properties of these materials, the failure mechanism and location of tensile and compressive testing commonly used in CFW [24,49] are not comparable.

**Figure 6:**
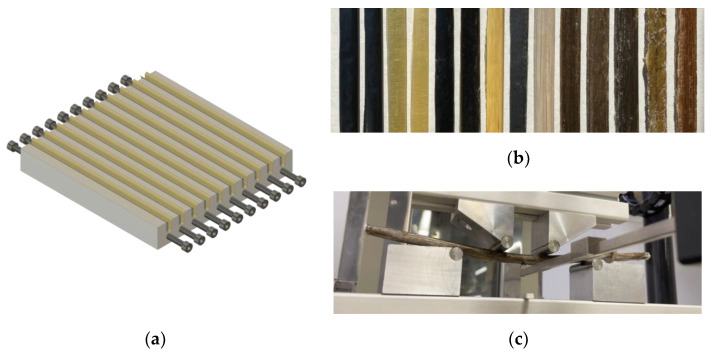
Four-point bending sample production and testing. (**a**) Aluminum mold for the sample production; (**b**) one produced sample of each material combination; (**c**) four-point bending test of flax sample.

As a remedy for this study, a specific sample with some geometrical adjustments (Figure 6) was designed to perform a four-point bending test in accordance to DIN EN ISO 14125 [50]. Compared to 3-point bending, the key benefit for this study is that load induction and failure position are separated. Through this method, the flexural stiffness and strength of the composite materials can be calculated and compared.

#### 2.3.1. Samples Geometry and Production

To maintain CFW fabrication characteristics but obtain comparable geometries, the samples had to be produced in a hybrid CFW process using an aluminum mold (Figure 6). The designed setup contained ten cavities to place the fibers and anchor elements at the sides for winding. During production, the samples lay on their side so that the mold walls defined the sample height. The cavities were filled until the upper edge was reached, setting the sample width. In this way, the sample height (structural depth) remained as constant as possible for all the samples, reducing the fluctuations in the flexural properties and making the samples comparable even though they presented a different number of filaments or weight. The cavity width (sample height) was set so that the material with the largest bundle diameter, the hemp fibers, fitted in at least once. The sample width was then set to a multiple of this value. Since the top and bottom of the samples needed to be plane-parallel, no draft angle was integrated. For demolding, a polytetrafluoroethylene (PTFE) foil was inlaid prior to winding.

The fiber materials were dried before processing, reducing the influence of moisture. The fibers were placed without crossings during the winding, except for the last layer, while counting the repetitions. After thermal curing of the resin, the bolts were unscrewed, the samples were removed from the mold and PTFE foil, and cut to length. Prior to testing, the sample dimensions as well as the weight were measured, and the average densities were determined. The sample volume can be measured via the outer geometry (cuboid) using a caliper or utilizing the Archimedes’ principle by weighing it in a liquid with a known density and temperature (Table 4).

The fiber bundles needed to fill the cavity spans from 2 for the hemp (H), and up to 300 for the aramid (A). This difference demonstrates the substantial variation in the linear density of the selected fibers. The average variation in sample height was only 6.5% of the one for the sample width. There was no correlation between the number of required bundles and the variation in cross-sectional dimensions, nor between the variation in the sample height and width. For example, the S-glass (G2) exhibited the highest deviations in the cross-sectional between samples, and the stainless steel (S) the lowest. The sample mass ranged from 22 g for the aramid (A) to 41 g for the stainless steel (S). Moreover, while the aramid showed the lowest deviations in mass between samples, the highest deviations were found in the basalt (B2), S-glass (G2), and hemp (H). The last two also entailed the highest deviations in both volume parameters. The average fluctuation in the geometrically determined volume was 16% higher than in the volume by Archimedes’ principle. The latter volume parameters averaged only 86% of the geometric volume ones, which is plausible since the geometric volume overestimated the actual sample value due to curvature on the sample edges.

#### 2.3.2. Composite Composition Calculation Methods

The inner composition of the samples must be determined for structural calculations and for an environmental impact assessment. The reference quantity for structural analyses is the volume or cross-sectional area of the sample, while for material consumption analyses, the relevant one is the mass. The presence of voids is reflected only in the volume-related variables, as such cavities that are filled with air, which is weight-neutral. The results are shown in the following parameters: fiber volume ratio (FVR), resin volume ratio (RVR), void volume ratio (VVR) as well as fiber mass ratio (FMR), and resin mass ratio (RMR). The volume and mass ratios can be converted using the densities of fiber and resin material by neglecting voids (Equations (1)–(4)):

(1) $$\mathrm{FVR}=\frac{\mathrm{FMR}\;\rho_{R}}{\mathrm{FMR}\;\rho_{R}+\left(1-\mathrm{FMR}\right)\;\rho_{F}\;},$$

(2) $$\mathrm{FMR}=\frac{\;\mathrm{FVR}\;\rho_{F}}{\rho_{R}\left(1–\mathrm{FVR}\right)+\mathrm{FVR}\;\rho_{F}\;},$$

(3) $$\mathrm{RVR}=\frac{\mathrm{RMR}\;\rho_{F}}{\mathrm{RMR}\;\rho_{F}+\left(1-\mathrm{RMR}\right)\;\rho_{R}\;},$$

(4) $$\mathrm{RMR}=\frac{\;\mathrm{RVR}\;\rho_{R}}{\rho_{F}\left(1–\mathrm{RVR}\;\right)+\mathrm{RVR}\;\rho_{R}}.$$

These conversions highly depend on the reliability of the densities of fiber $\rho_{F}$ and resin $\rho_{R}$. In the case of natural fibers, this is not trivial due to the variations of the material parameters along the fiber. Some fibers are also hollow, making it more difficult to obtain representative densities. A pyrolysis [51] is not possible for the natural fibers due to their low-temperature resistance. Voids cannot be neglected in CFW due to the absence of a consolidation process step. Thus, mass- and volume-related parameters must preferably be determined separately. With natural fibers, averaging parameters is necessary. Besides, the mean or median can be more meaningful in different situations depending on the distribution. If the micrographs indicate that hollow fibers have been compacted during the process, this should also be considered in the parameters. The FVR can be determined without any conversion by:

(5) $${\mathrm{FVR}}_{\left(\mathrm{area}\right)}=\frac{\pi \;d^{2}\;f\;r}{4\;a},$$

with

*d* filament/fiber diameter,
*f* number of filaments/fibers per roving/fiber bundle,
*r* number of rovings/fiber bundles,
*a* relevant cross-sectional area of the sample.

If the filament diameter is unknown, it can be measured from microsections or by a micrometer screw, especially for thicker filaments. In case the number of filaments/fibers is missing, it can be calculated by:

(6) $$f=\frac{4\;T}{\pi \;d^{2}\;\rho_{F}},$$

with

*T* linear density of the roving/fiber bundle.

The number of rovings at a certain location in the fiber net can be identified by the winding plan [14]. It can also be counted from the microsections, if the individual bundles are distinguishable by resin-rich interfaces [9]. The sample cross-sectional area can be measured using a caliper as the product of the sample height, width, and a form correction factor [14]. This value can also be retrieved from microsections [52]. Another option is to calculate the FMR by:

(7) $${\mathrm{FMR}}_{\left(\mathrm{mass}\right)}=\frac{m_{F}}{m_{S}}=\frac{T\;r\;l}{m_{S}},$$

with

$m_{F}$ fiber mass per sample, as total fiber length deployed during production,
$m_{S}$ sample mass,
*l* sample length,

and then to convert it via Equation (1). This method entails the uncertainties of the fiber and resin densities. The linear density can be determined by measuring the length and weighing of the dried roving/fiber bundle. The sample mass can be measured on a scale if the material is equally distributed over the relevant section of the component. There are also alternative methods to get the FMR via the sample volume as:

(8) $${\mathrm{FMR}}_{\left(\mathrm{vol}/\mathrm{den}\right)}=\frac{m_{S}-\rho_{R}V_{S}}{V_{S}\left(\rho_{F}-\rho_{R}\right)},$$

with

$V_{S}$ sample volume.

This method may be beneficial if the cross-sectional parameters are more complicated to determine than the total sample volume, which can be calculated from geometry $V_{S\left(\mathrm{vol}\right)}$ or by deploying Archimedes’ principle $V_{S\left(\mathrm{den}\right)}$. The RMR can be calculated as:

(9) $${\mathrm{RMR}}_{\left(\mathrm{mass}\right)}=\frac{m_{S}-m_{F}}{m_{S}},$$

with

$m_{R}$ resin mass per sample, as resin absorbed by the component during fabrication,

and then converted into the RVR using Equation (3). Fiber and resin consumptions can also be read from the fabrication protocol, which should include the weight of the fiber bobbins and resin containers before and after the winding. This method is only useful if resin leakage can be neglected and fiber cutoffs are absent or logged. The VVR can then be calculated as:

(10) $${\mathrm{VVR}}_{\left(\mathrm{area}\&\mathrm{mass}\right)}=1-{\mathrm{FVR}}_{\left(\mathrm{area}\right)}-{\mathrm{RVR}}_{\left(\mathrm{mass}\right)}.$$

By using the FVR calculated via the cross-sectional area (Equation (5)), fewer uncertainties are incorporated. Alternatively, the VRR can also be obtained via the density of the sample as:

(11) $${\mathrm{VVR}}_{\left(\mathrm{den}\right)}=\frac{\rho_{C}-\rho_{S}}{\rho_{C}},$$

with

$\rho_{C}$ theoretical compressed sample density,
$\rho_{S}$ sample density.

The theoretical compressed samples density is given by:

(12) $$\rho_{C}=\frac{\rho_{F}\;\rho_{R}\;\left(m_{F}+m_{R}\right)}{\rho_{F}\;m_{R}+\rho_{R}\;m_{F}}.$$

**Figure 7:**
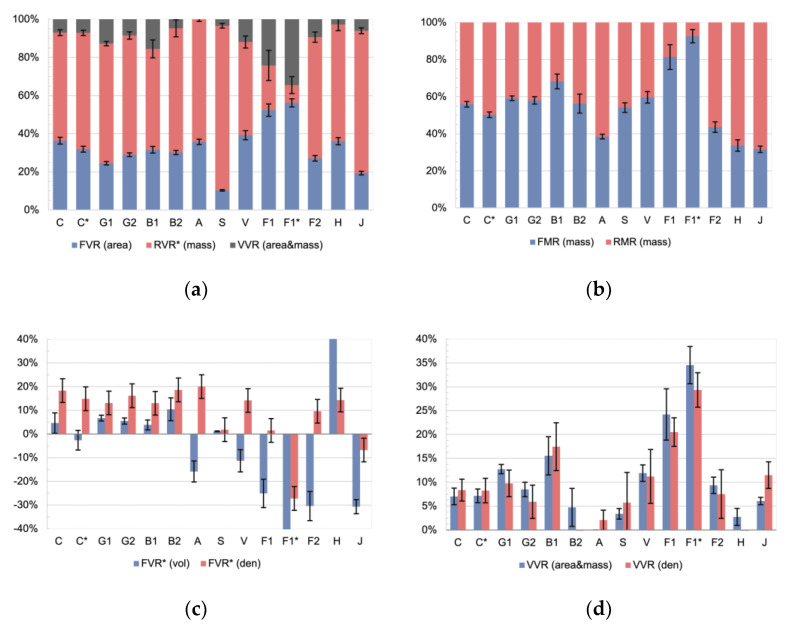
Overview of the inner composite composition of the four-point bending test samples, values marked with an asterisk include a conversion via the density-depended Equations (1)–(4) (**a**) volume ratios; (**b**) mass ratios; (**c**) FVR obtained by volume and density measurements (Equation (8)), with non-plausible values; (**d**) void ratios in comparison.

Applying these equations to the produced four-point bending samples results in the following characteristic values (Figure 7).

The internal composition of the samples can be given with respect to the sample volume (Figure 7a) or mass (Figure 7b). The void content is included in the volume-related data but not in the mass-related data. The FVR _(area)_ for the technical fibers C–A was in the range between 38–25%, whereas the value for steel was at 10%. Based on previous experience in CFW on carbon and glass fibers, this value is remarkably low, especially the one for steel. The hybrid winding technique used in this study can be identified as the main contributor to this decreased FVR _(area)_ values. The mold prevented the drainage of resin during the winding and curing stages. For the natural fiber types V–J, the FVR _(area)_ covered a comparatively larger range, between 19–56%. The scatter between samples of the same fiber type was 62% larger on V–J compared to the technical fibers. The natural fibers incorporated higher fluctuation in the material parameters. Interestingly, the highest FVR _(area)_ was achieved in the flax tape, which also had the highest values for the VVR _(area&mass)_. Since the RVR* _(mass)_ was converted via Equation (3), it showed the higher standard deviation of all materials. The FVR _(area)_ of the flax yarn sample was lower than the one of the tape and both were to be expected. The VVR _(area&mass)_ for the aramid was calculated to be −6.2%, which as a negative value is not plausible. It can be assumed that the VVR for the aramid was very low due to the comparatively high number of rovings per sample. For carbon, the petroleum-based resin samples showed higher values in the FVR _(area)_ and the FMR _(mass)_ compared to the respective bio-based sample sets. This ratio was reversed on the flax tape samples.

When considering the ratios related to mass (Figure 7b), the same trend was found in the data regarding both the absolute FMR _(mass)_ ratios and their standard deviations; however, the FMR _(mass)_ values were higher, between 32–93%, which can be explained by the ratio of the densities between fibers and resins. Obviously, very high values such as those for the flax tape should be used with caution. It can be noticed that the outlying value for steel is no longer present in the mass-related analysis. Compared to the technical fibers, the natural fibers V–J showed, on average, a 46% larger standard deviation between samples of the same fiber type.

Both methods using the sample volume as an input (Equation (8)), become useless when applied to the natural fibers (Figure 7c). For the technical fibers C, G1/2, B1/2, the method relying on the geometrical volume returns plausible values, and the method using Archimedes’ principle also works for C* and A. As expected, the FVR* _(vol)_ strongly underestimates the real value, as the sample geometry is assumed to be a cuboid. The FVR* _(den)_ is used instead since typical CFW geometries cannot be well approximated using cuboids. Compared to the FVR _(area)_, the FVR* _(den)_ underestimated the ratio on average by 49%. The standard deviation on the FVR* _(den)_ ratios was compared to the area-based volume ratios (Figure 7a) 4.2 times higher and compared to the mass ratios (Figure 7b) 2.3 times higher. Therefore, area-based and mass-based methods are preferable to use over volume-based approaches.

Although the ratios calculated by volume are not reliable, there was a good match for all samples except B2, A, and H when comparing the VVR _(area&mass)_ and the VVR _(den)_ (Figure 7d). The VVR _(den)_ scattered 76% more than the VVR _(area&mass)_ if averaged over all material types.

In conclusion, for volume-based calculations, the FVR _(area)_ should be used and for a calculation referring to mass, the FMR _(mass)_ should be used instead. This differentiation avoids including the uncertainty of the density-related conversion equations. Area- and mass-related methods should be preferred over volume-based methods, especially when applied to natural fibers.

## 3. Results

### 3.1. Fabrication Suitability

**Figure 8:**
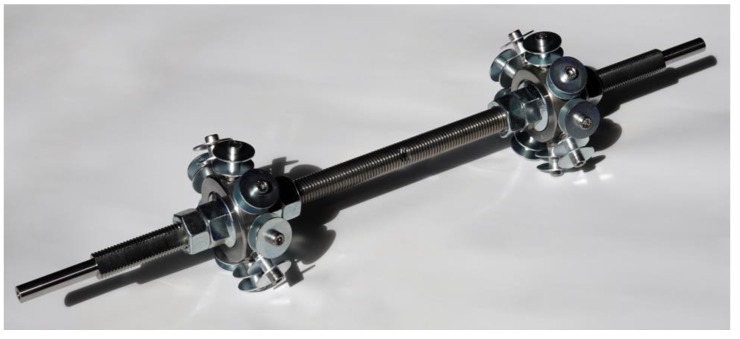
Winding fixture for the generic cylindrical samples equipped with medium size winding pins.

Generic cylindrical components were coreless wound to test the fabrication suitability of the various selected materials using the developed equipment. The winding fixture (Figure 8) for those samples consisted of two metallic adapters whose distance could be adjusted by nuts on a threaded rod. Each adapter held nine equally distanced winding pins. The holes for the winding pins were at an angle matching the average of the possible fiber arrangements defined by the syntaxes. This tilt of the winding pins in relation to the components’ main axis prevented fiber kinks on the washers of the winding pins. This winding fixture allowed the production of samples with a regular pattern with three different configurations. The three different winding syntaxes were created by a relative rotatory shift of 1–3 winding pins when changing sides during the winding. The threaded rod could be clamped at the ends to support this rotation. A shift of four winding pins was not possible since the fibers would have touched the threaded rod.

The winding syntax of the samples started with a sine–cosine-shaped edge reinforcement, followed by a fiber bridge connecting both sides, and the repetition of the edge reinforcement sub syntax on the other side. Then, the sub syntax followed that formed the component. Here, after each hooking, the side was changed with the shift between winding pins, and the fixture was rotated in the traveling direction to maintain the working area. At the end of this sub syntax, another edge reinforcement per side was wound connected by a second fiber bridge. The edge reinforcements sub syntaxes could be carried out several times, especially for fibers with a low linear density. After curing the resin, the fixture could be disassembled, and the sample removed.

The switch from petroleum-based to bio-based epoxy resin did not impact the winding equipment. Although an increase in resin leakage must be expected with a decrease in viscosity, this was not the case with the bio-based resin. The sample size and the consequential duration of a winding session were not big enough for the pot life to make a difference. The only noticeable impact of using the bio-based resin was that curing could not take place at room temperature. During the curing of the bio-based resin in the oven, its viscosity dropped before the crosslinking process was sufficiently advanced to prevent the resin from flowing down due to gravity. Thus, samples accumulated resin on the side that was facing downwards. The thicker the fiber bundle, the more pronounced this effect became. In extreme cases, resin dripping from the component could also be observed, which increased the FVR of the component locally during curing. The uneven distribution of the resin can be detected by measuring the cross-sectional area of the fiber bundles along the sample. An uneven resin distribution has a negative impact on the structural performance of such a component. Rotating the sample during curing could reduce this effect.

For each fiber material processing, the ceramic eyelets and the setting of the ceramic ring in the winding head had to be adjusted. The setting with the lowest deflection possible at sufficient impregnation of the fiber bundle core was selected.

**Figure 9:**
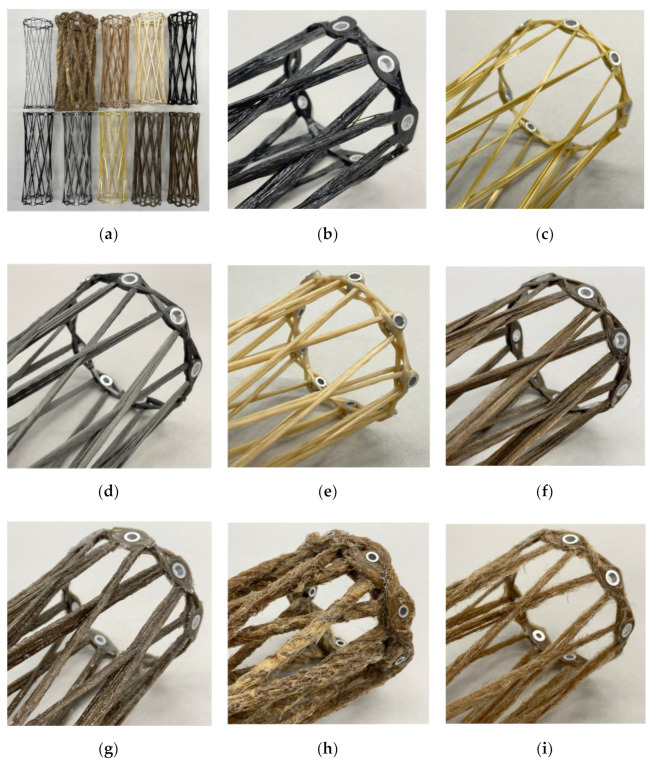
Selection of some of the produced generic cylindrical samples, from left to right and top to bottom: 1 × 6k carbon, hemp, jute, viscose, basalt type 1, 3 × 24K carbon, stainless steel, aramid, flax yarn, and flax tape. (**a**) Size comparison overview; (**b**) basalt; (**c**) aramid; (**d**) stainless steel; (**e**) viscose; (**f**) flax tape; (**g**) flax yarn; (**h**) hemp; (**i**) jute.

Carbon (Figure 9a) and glass exhibit excellent processability as the CFW winding process was developed using these two fiber materials. This was also confirmed with the winding head developed in this study with the glass fiber filaments breaking more easily on the fiber guide elements compared to the carbon fiber filaments. Both basalt fiber types (Figure 9b) performed similarly to the glass fiber types. The aramid fibers (Figure 9c) presented comparable handling characteristics to the carbon fibers, although resin dripping from the free-spanning impregnated fiber bundle was observed. The deployed stainless-steel roving (Figure 9d) had low internal cohesion, which considerably complicated the damage-free handling and processing. The reason for this was the lack of sizing. When applying a compressive load, the roving fanned out. This disadvantageous behavior was not observed with any other selected material.

With a setting with a lower deflection, the viscose roving (Figure 9e) was not well impregnated in the core of the roving. Therefore, a higher deflection setting was selected. The viscose material also showed less coherence between layers, similar to the flax yarn. The flax tape (Figure 9f) showed a better impregnation compared to the flax yarn (Figure 9g). This improvement resulted from the shorter distance between the core and the contour of the flax tape. With a circular yarn, the resin filtering effect of the dry fiber material was more pronounced. In addition, the flax yarn had a twist which also contributed to this effect on the impregnation. Another effect of the tape was that a better bonding between the laminate layers could be achieved because of the larger contact surface; however, in order to benefit from this, the rotational orientation of the tape during winding must be consciously controlled. If this is not the case, unwanted changes in fiber alignment can occur, which can cause the tape to fold. This consequence would significantly reduce the structural performance. The flax tape showed no hairiness, whereas the hairiness was present in the yarn and had a perceptible effect on the CFW process at a tested winding duration of up to one hour.

Due to the high linear density of hemp (Figure 9h), major adjustments to the impregnation units were necessary. The ceramic eyelets were replaced by a larger metallic insert connected to the cartridges’ cut cone via a thread. The ceramic ring had been removed. Due to the thickness of the card sliver, impregnation took an equivalent amount of time, which noticeably reduced the winding pull-off speed. The crocheted yarn around the hemp caused strong local diameter variations along the contour of the fiber bundle, but it did not cause any issues during fabrication. The strongly pronounced hairiness was remarkable in the jute yarn (Figure 9i). The hairiness led to a strong accumulation of fiber fragments in the winding head, which the sponge retained. As a consequence, the length of individual winding sessions was limited.

In this study, except for the flax fibers, all selected natural fibers showed a significantly higher resin consumption per sample than the benchmark fibers, carbon and E-glass. When scaling up this CFW process with natural fibers to an architectural scale, two further general process characteristics would apply to any fiber material with lower mechanical properties, namely, the free-spanning fiber length and the fiber tension during winding should both be limited. The tension should preferably be actively controlled since tension peaks exceed the ultimate strength of the weaker material quicker. Unanticipated fiber rupture is a safety hazard to personnel, which usually happens at the winding pin or the winding head nozzle. By limiting the distance between the TCP and the last winding pin, sagging at low fiber tension levels can be avoided.

### 3.2. Structural Performance

**Figure 10:**
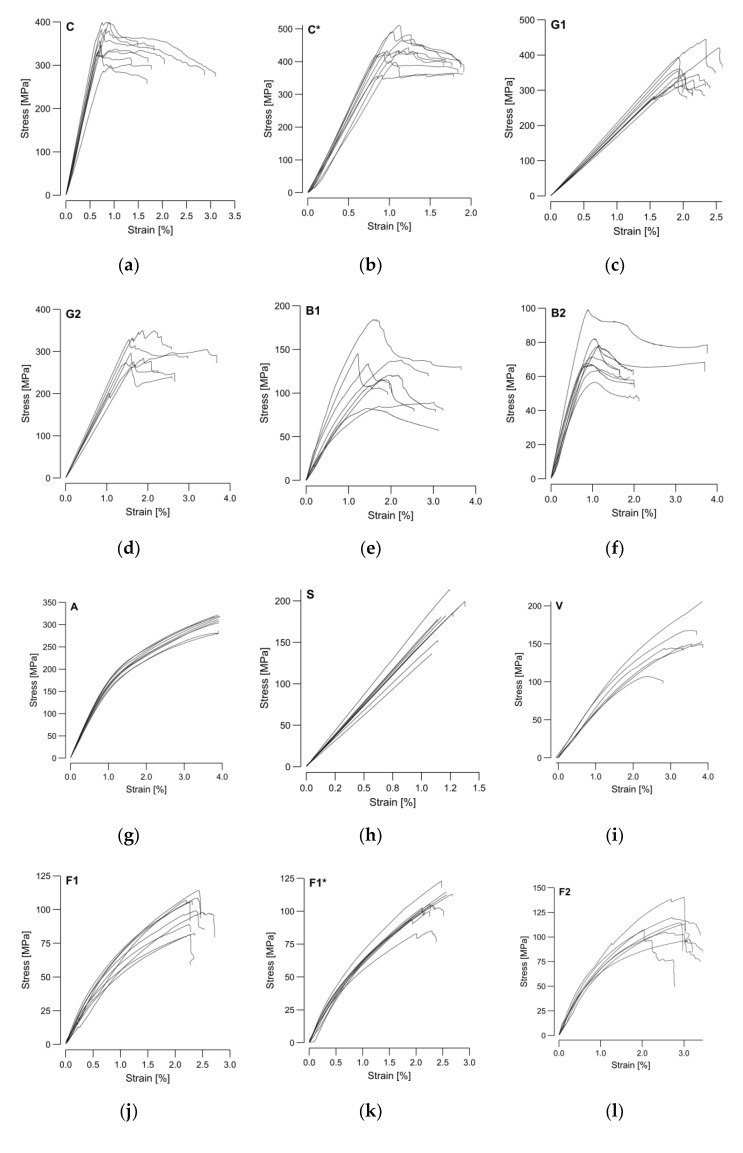

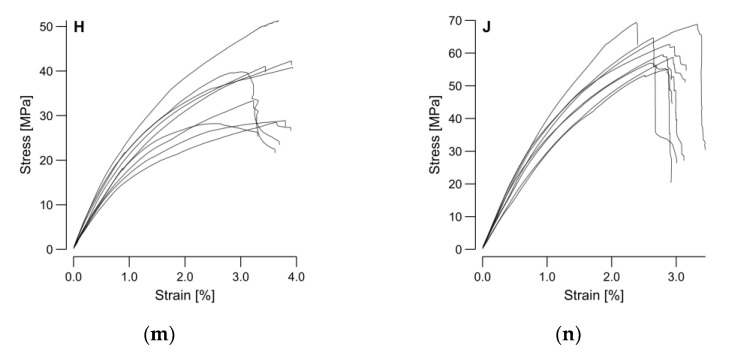
Stress–strain diagrams of the four-point bending tests for each induvial sample set. (**a**) Carbon—C; (**b**) carbon with petroleum-based resin—C*; (**c**) E-glass—G1; (**d**) S-glass—G2; (**e**) basalt, type 1—B1; (**f**) basalt, type 2—B2; (**g**) aramid—A; (**h**) stainless steel—S; (**i**) viscose—V; (**j**) flax tape—F1; (**k**) flax tape with petroleum-based resin—F1*; (**l**) flax yarn—F2; (**m**) hemp—H; (**n**) jute—J.

**Figure A1:**
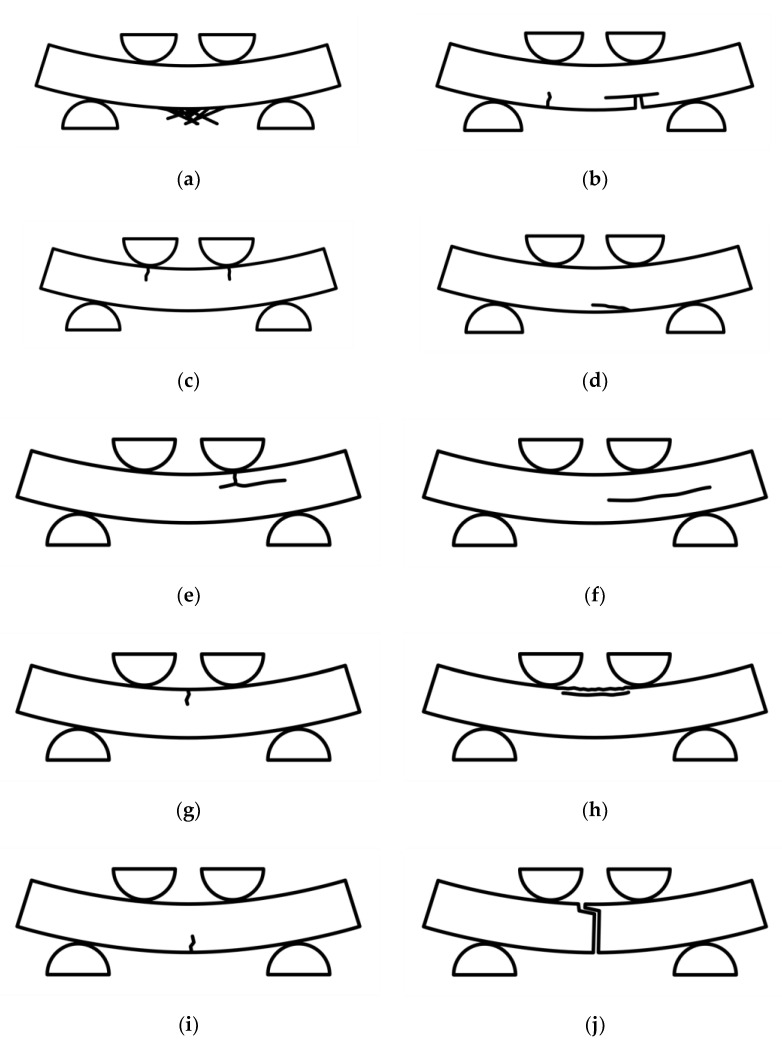
Failure modes in the four-point bending tests. The naming of the failure type matches the label of the subfigure. (**a**) Fiber fracture triggered by tensile stresses resulting in fibers sticking out of the sample surface in the lower center; (**b**) matrix cracking and local fracture triggered by tensile stresses opposite to the upper load induction; (**c**) matrix cracking and local fracture triggered by compression at the upper load induction points; (**d**) fracture triggered by interlaminar shear and tensile stresses at the bottom center; (**e**) fracture triggered by interlaminar shear and compression stresses at the top; (**f**) fracture triggered by interlaminar shear stresses at the sample middle; (**g**) matrix cracking and local fracture triggered by compression at the upper center; (**h**) fracture triggered by compression at the surface between the upper load induction points; (**i**) matrix cracking and local fracture triggered by tensile stresses in the lower center; (**j**) fracture of the whole sample triggered by tensile stresses in the lower center.

As described in Section 2.3, the four-point bending test samples were manufactured and tested to compare the materials’ structural performance and support the subsequent sustainability assessment. The force-displacement measurements of the universal testing machine were converted into stress–strain curves (Figure 10) using the measured sample height and width from Table 4 and applying large-deformation equations for the bending test [50]. It can be observed that the scatter between the stress–-strain curves was more pronounced for the natural fibers. The samples failure modes were classified based on the types in Figure A1.

Compared to the other investigated materials, the stainless-steel samples showed the most brittle failure behavior. All steel samples (S) exhibited a complete fracture into two or three pieces, triggered by tensile stresses in the lower center of the sample (type J—Figure A1j). Carbon, glass, and steel presented a clear elastic behavior range. The failure behavior of carbon (C and C*) and E-glass (G1) was dominated by fracture triggered by compression at the upper load induction points (type C—Figure A1c) occasionally mixed with fracture triggered by tensile stresses at the sample’s bottom (type B—Figure A1b). The failure modes on S-glass (G2) were less consistent, as they also included sporadically types G, H, and I (Figure A1). Aramid and basalt also exhibited a distinguishable elastic zone, but they did not present a clear yielding point. The prominent failure type on the aramid samples was a fracture triggered by compression at the surface between the upper load induction points (type H), with a rare occurrence of types C and F. One of the basalt sample types (B1) showed similar failure behavior to the E-glass. In contrast, the other basalt (B2) also entailed failure types I and G.

**Figure 11:**
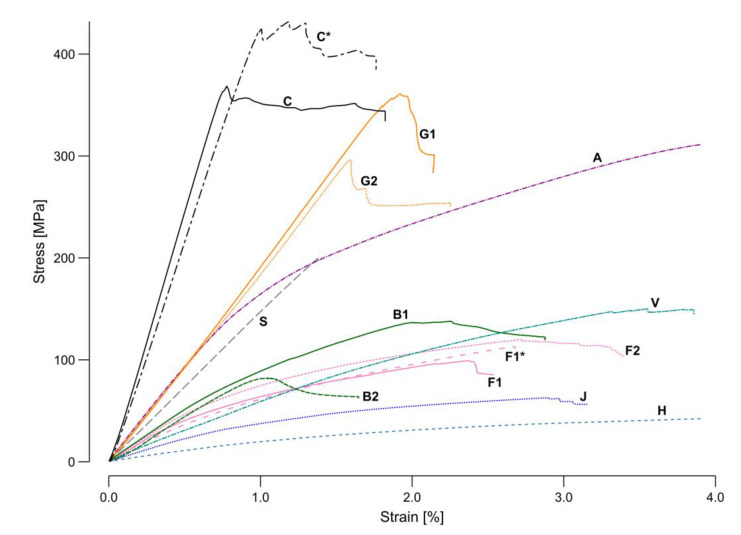
Comparative scaled stress–strain diagram of the four-point bending tests by selecting a representative graph of each sample. C—carbon, C*—carbon with petroleum-based resin, G1—E-glass, G2—S-glass, B1—basalt, type 1, B2—basalt, type 2, A—aramid, S—stainless steel, V—viscose, F1—flax tape, F1*—flax tape with petroleum-based resin, F2—flax yarn, H—hemp, J—jute.

In contrast to the mentioned technical fibers, the behavior of the natural fibers was mostly ductile with large areas of strain hardening. Only basalt (B1 and B2) and the flax yarn (F2) also presented necking areas before failure. Whereas the flax tape (F1) was dominated by failure type D (isolated with A, B, C, and I), the usage of the petroleum-based resin with flax (F1*) changed the dominant failure type to B (isolated with D, A, and I). The flax yarn (F2) showed more variable failure behaviors with type B combined with C, I, and A. Whereas the jute (J) failed by type B and viscose (V) by type C, the failure type on the hemp samples (H) was not identifiable. The variations in the manufacturing process caused the failure deviations in the same sample type, whereas between the different materials the ratios in the mechanical properties of the fiber and resin were decisive. Figure 11 presented a single averaged sample per material to provide a direct, scaled comparison between the different investigated materials.

**Figure 12:**
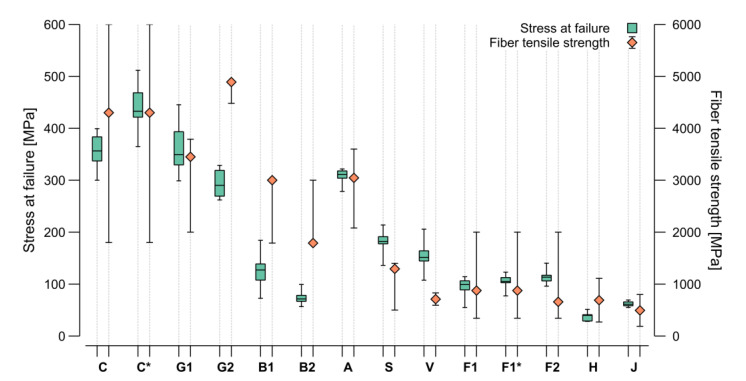
Comparison between the stress at failure (green) in the four-point bending tests and the fiber tensile strength (orange) of the selected product, with its placement in the range reported in literature. For H and J, the average values were calculated from the literature. The whiskers for both data traces represent the min/max range. Scale factor between axis is 1:10. C—carbon, C*—carbon with petroleum-based resin, G1—E-glass, G2—S-glass, B1—basalt, type 1, B2—basalt, type 2, A—aramid, S—stainless steel, V—viscose, F1—flax tape, F1*—flax tape with petroleum-based resin, F2—flax yarn, H—hemp, J—jute.

**Figure 13:**
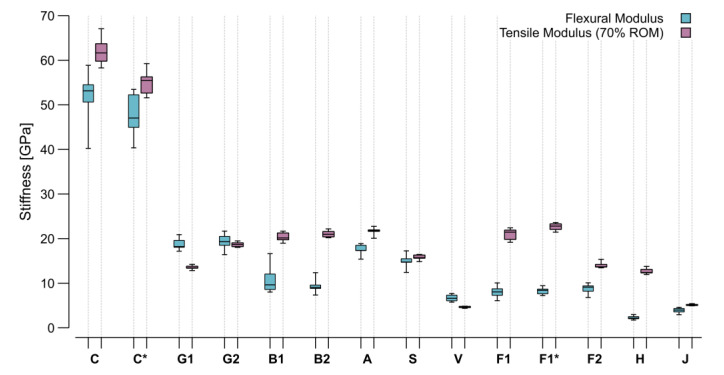
Comparison of the flexural modulus obtained from the four-point bending test and 70% of the tensile modulus calculated by the rule of mixture (ROM). The whiskers of the boxplots represent the min/max range. C—carbon, C*—carbon with petroleum-based resin, G1—E-glass, G2—S-glass, B1—basalt, type 1, B2—basalt, type 2, A—aramid, S—stainless steel, V—viscose, F1—flax tape, F1*—flax tape with petroleum-based resin, F2—flax yarn, H—hemp, J—jute.

Carbon and flax tape were investigated with both the bio-based and the petroleum-based resin. A higher ultimate strength could be found for the petroleum-based resin in both cases (Figure 11). This effect was more prominent in the carbon samples; however, in terms of stiffness, the bio-based resin with carbon showed higher values. Based on this graphical representation of the test data, four clusters could be identified. The group with the highest performance included both carbon samples. The next group was formed by glass, aramid, and stainless steel. The third group entailed basalt, viscose, and flax. In the least performing group, jute and hemp could be found. The characteristic mechanical performance values (strength and stiffness) were extracted from the stress–strain curves and plotted in Figure 12 and Figure 13 for comparison with theoretical values.

Figure 12 shows a comparison of the stress at failure, as the highest load achieved in the four-point bending test (green), and the fiber tensile strength (orange), highlighting the ratios between the different fiber materials. The resin amount in the composite described by the FVR (Figure 7) plays an important role in the composite strength, as this depends on the ratio between the two materials. In general terms, the more resin oversaturation, the less strength, with this effect being more remarkable with high-strength fibers. The orange markers in the graph (Figure 12) show the placement of the fiber products used for the test within the min-max range reported in the literature. For the carbon, aramid, and viscose, a product in the middle of the range was selected. Glass and steel were in the upper part of the range, while the flax was in the lower one. For the basalt, two products were selected at the extremes of the range reported in literature. For the hemp and jute, the average from the literature is represented as no manufacturer values were provided. It can be expected that the ratio between the fiber strength and the samples stress at failure was maintained; however, some deviation can be explained either because of a low FVR (Figure 7) or due to other fabrication issues. The lowest FVR _(area)_ was observed in the steel, jute, flax tape, and E-glass. All these materials performed relatively better than their datasheet value would predict. Although the highest VVR _(area&mass)_ was found in the flax tape and basalt, no performance anomalies reducing the stress at failure could be found.

In terms of impregnation, the hemp, viscose, and flax yarn performed worst during the fabrication suitability testing. This detriment did not translate into a decrease in stress at failure for the flax yarn and the viscose; however, the values for the hemp were on the lower end of the range reported in the literature. These results may be related to the fabrication or the material’s appearance as a crocheted card sliver. Thus, a high RVR* _(mass)_ did not equal to good impregnation. Having a similar FVR _(area)_, both versions of the carbon samples (C and C*) and of the flax tape (F1 and F1*) should have similarly or slightly better performed, as the bio-based resin was higher in strength than the petroleum-based resin (Table 3); however, the achieved strength was lower in both cases, concluding that the bonding between the fiber and bio-based resin was still not as good as with the petroleum-based resin. The loss in performance was less on the flax compared to the carbon, which could have resulted from the lower viscosity of the bio-based resin achieving a better impregnation on the flax, an effect that did not seem relevant for the carbon. The bonding between the individual fiber bundle layers did not seem to affect this testing scenario. The viscose and flax tapes exhibited the worst bonding, but both performed better than the expectation coming from their datasheet values.

The overall impact of the bio-based resin was less influential than the selection of the fiber product along the range reported in the literature. Based on the carbon and flax samples, a reduction of about 10–20% was observed in the stress at failure when switching from a petroleum-based to a bio-based resin; however, changing the fiber product resulted in a 20–70% shift and was thus more influential. The difference between theoretical values and measured performance were more prominent in the S-glass sample (G2) and both basalt types (B1 and B2), where the behavior trend was not maintained, and the results showed a significantly lower performance than in the literature. The measured performance of viscose and steel fibers was also outside the expected range but from the upper bound. These results show that it is not reliable to fully trust data sheet information for CFW. In all cases, deviations have to be attributed to fabrication issues. It is remarkable that the interquartile ranges of the boxplots in relation to the length of their whiskers were larger on the technical fibers, showing the difference in uncertainties between both material groups.

The Voigt estimate or rule of mixtures (ROM) can approximate the longitudinal tensile modulus ($E_{T}$) of unidirectional composites by assuming that the resin and fibers deform together [53]. Most recent CFW full-scaled structural tests [12,16,24] have shown a reduction to 70% of the ROM, as:

(13) $$E_{T}=0.7\;\left[E_{f}\;\mathrm{FVR}+E_{m}\left(1-\mathrm{FVR}\right)\right],$$

with

$E_{T}$ tensile modulus of the composite,
$E_{f}$ tensile modulus of fibers,
$E_{m}$ tensile modulus of matrix,

giving appropriate results for initial assumptions of the bundles’ tensile modulus. This conservative reduction is necessary because the CFW structures entail voids that prevent the bundles from properly bonding, which is one of the necessary assumptions to apply to the ROM. Moreover, deviations in the FVR along the same sample can create deviations in the real stiffness of the specimen. The flexural modulus calculated from the bending test was expected to be slightly lower than the tensile modulus, but it should have shown the same trends. The calculated ROM was based on the datasheet values for the material (Table 3) and took into account the FVR _(area)_ (Figure 7). Therefore, the ROM ratios between the different materials should also have been kept by the four-point bending test flexural modulus. Appling the ROM allowed for comparing the test stiffness results and datasheet values with the same units under consideration of the measured FVR _(area)_ influence (Figure 13).

There were several samples where the results were remarkably lower than the calculated ROM, such as both basalt types, which already showed the same detrimental behavior with the stress at failure (Figure 12). All the flax samples (F1, F1*, F2) also showed a lower stiffness than predicted. This difference was smaller on the flax yarn compared to the flax tape. The tape had a low dry fiber strength at break due to its manufacturing method, which created cohesion by stapling the filaments. Consequently, the strength of longer tape sections relied on fiber-to-fiber friction rather than the actual strength of the fibers. As the FVR _(area)_ and VVR _(area&mass)_ of these samples were remarkably high (Figure 7), the low strength between the individual fibers could have translated directly into a lower sample stiffness. This behavior of the tape flax fibers has already been observed in previous CFW applications [24].

Similar to the overperforming of the viscose in strength, its stiffness value was also higher than predicted. Both glass types also showed a slightly higher measured stiffness than that calculated by applying the ROM. Despite the steel samples’ remarkably low FVR _(area)_, the results matched the prediction in this case. The comparison in stiffness between both resin types used, did not show a significant difference; however, a decrease could have been predicted based on the resin material properties (Table 3).

**Figure 14:**
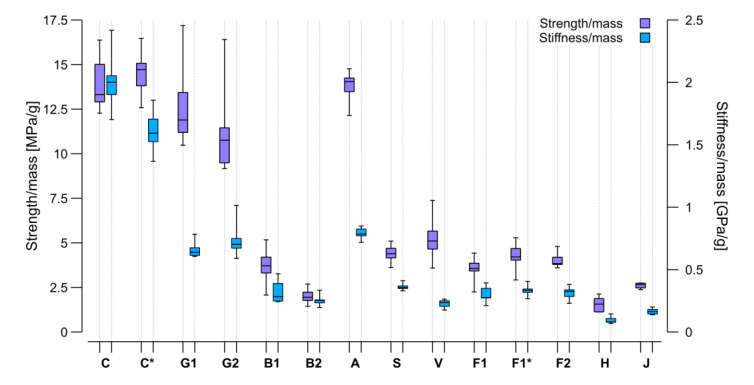
Comparison of the strength and stiffness per mass of each sample type. Axes are differently scaled. The whiskers of the boxplots represent the min/max range. C—carbon, C*—carbon with petroleum-based resin, G1—E-glass, G2—S-glass, B1—basalt, type 1, B2—basalt, type 2, A—aramid, S—stainless steel, V—viscose, F1—flax tape, F1*—flax tape with petroleum-based resin, F2—flax yarn, H—hemp, J—jute.

As the absolute stiffness and strength values from the four-point bending test were extracted, the next step was normalizing the results based on the sample mass and obtaining a statement on the suitability of the materials for lightweight constructions (Figure 14). Among the masses of the different sample types measured (Table 4), the aramid samples were found to be the lightest and steel the heaviest.

Due to its lightweight character, the aramid gained performance in the comparison of the strength/mass ratio, which made it comparable to the carbon and better than both glass types. This result is attributable to the high toughness of aramid. Similar behavior can be found on glass. It is remarkable that despite its relatively high density, the glass sample mass did not increase. Jute, being the second lightest of all, exhibited strength and stiffness comparable to other natural materials and performed even better than hemp. As steel was by far the heaviest material in the selection by fiber density and sample mass, the values obtained for its samples decreased in performance in this comparison. The basalt and viscose also reached lower performance ratios due to their sample weights. This resulted in other materials that presented a lower strength and stiffness having higher mass-specific performance values, as was the case for all the flax types.

Considering all these facts, the carbon still presented the best ratios regarding both strength and stiffness per mass unit; however, the glass and aramid were comparable in terms of strength. Viscose presented a good strength to mass ratio among the natural fibers, but the flax balanced stiffness, strength, and mass the most equally. The results described in Figure 13 and Figure 14 show that the flax could have an even higher stiffness with an improved load transfer between individual fibers. Moreover, another product selection with higher overall mechanical properties, as described in the literature, is also possible. In addition, the petroleum-based resin also lowered the stiffness of the carbon samples while the flax yarn exhibited slightly higher mass-specific performance characteristics than the tape.

### 3.3. Sustainability Assessment

A comparison between the samples based on two representative sustainability markers was carried out. Such mass-specific comparison is only valid if the samples show the same mechanical properties and an ecological assessment is valid for a shorter period compared to an evaluation of mechanical parameters. This and other limitations are further discussed in Section 4.1.

The design of the samples aimed to keep their dimensions constant. Fabricating the samples with an adjusted cross-section to achieve the same weight or to perform equally in the four-point bending test was not feasible. Instead, linear behavior in the bending properties was assumed, and only the structural depth of the theoretical samples was incremented until each material matched the same force or stiffness of the reference sample, set as the bio-based resin and carbon (C). This approximation resulted in two cases. In Case 1, the structural depth was calculated for each sample so that it would fail at the same force level as the average carbon (C) sample. The formal relation describing this is:

(14) $$h_{S}=\sqrt{\frac{F_{C}\;L}{\sigma_{F,S}\;b_{S}}},$$

with

$h_{S}$ structural depth (sample height in Table 4),
$F_{C}$ maximum average force of the carbon fiber samples (C) at failure,
*L* length between supports in the bending test,
$\sigma_{F,S}$ sample flexural strength,
$b_{S}$ sample width (Table 4).

In Case 2, the structural depth was calculated for each sample so that it exhibited the same spring stiffness as the force over the strain at failure. Which expresses to:

(15) $$h_{S}=\sqrt[{3}]{\frac{k_{C}\;L^{3}}{4\;b_{S}\;E_{F,S}}}\;;\;k_{C}=\frac{F_{C}}{D_{C}}\;\;$$

with

$k_{C}$ spring stiffness of the carbon sample (C), as a function of the force ($F_{C}$) divided by the deformation ($D_{C})$ at failure,
$E_{F,S}$ flexural modulus of the samples,
$D_{C}$ carbon sample (C) maximum deformation at failure.

The method can be used to understand the relative performance and how these scaling factors could be used for design, as structural depth adjustments are common practice in structural engineering to increase both strength and stiffness.

**Figure 15:**
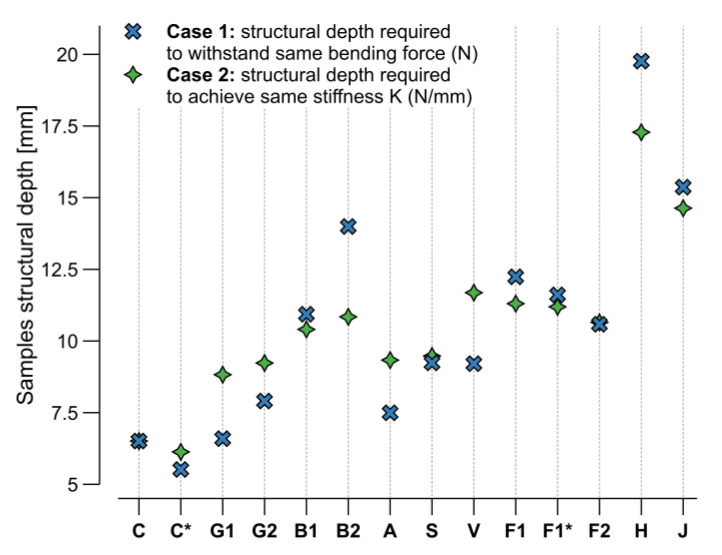
Theoretical sample structural depth of equally performing samples for both cases, Case 1 (strength) and Case 2 (stiffness). C—carbon, C*—carbon with petroleum-based resin, G1—E-glass, G2—S-glass, B1—basalt, type 1, B2—basalt, type 2, A—aramid, S—stainless steel, V—viscose, F1—flax tape, F1*—flax tape with petroleum-based resin, F2—flax yarn, H—hemp, J—jute.

Figure 15 shows the resulting structural depths for each material to either withstand the same bending force $F_{C}$ as carbon (Case 1) or achieve the same stiffness $k_{C}$ (Case 2). Except for C*, all the other samples required higher structural depths, and therefore, their final material amount increased respectively to be compared in the next step. Higher material amounts needed to support the same load would also result in a higher demand in the winding pin capacity, directly proportional to the values presented in Figure 15. When utilizing natural fibers, this issue could translate to the usage of larger winding pins, more winding pins, or different design strategies for the CFW process.

**Figure 16:**
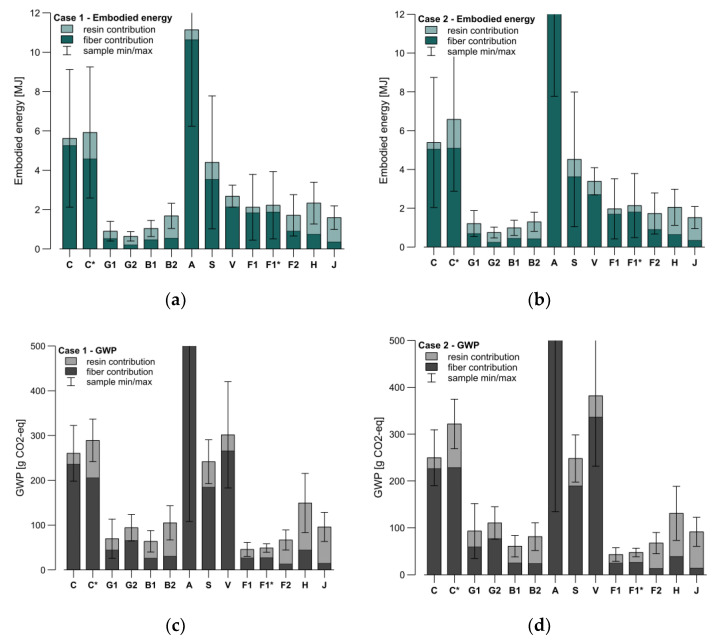
Sustainability assessment for theoretically equal-performance samples, Case 1 (strength) or Case 2 (stiffness) as per Figure 15. The contribution of the resin is stacked on top of the fiber contribution. Whiskers represent the samples’ deviations covering their full min/max range. C—carbon, C*—carbon with petroleum-based resin, G1—E-glass, G2—S-glass, B1—basalt, type 1, B2—basalt, type 2, A—aramid, S—stainless steel, V—viscose, F1—flax tape, F1*—flax tape with petroleum-based resin, F2—flax yarn, H—hemp, J—jute. (**a**) Embodied energy for Case 1; (**b**) embodied energy for Case 2; (**c**) global warming potential for Case 1; (**d**) global warming potential for Case 2.

For the sustainability assessment, each sample’s theoretical resin and fiber consumptions in grams were calculated based on the adjusted structural depth (Figure 15), the real sample width and length (Table 4), and the FMR (mass) (Figure 7). Then, both the embodied energy and GWP were calculated for each sample’s fiber and resin contribution in Cases 1 and 2 (Figure 16). This calculation used the average numbers found in the literature (Table 1 and Table 3).

As the values used for these calculations do not correspond to the actual products but are just taken as a reference from the literature, the graphs’ whiskers in Figure 16 represent the possible variation in the samples if their contributions were calculated with the maximum and minimum values found in the literature. These variations are the sums of the fiber and resin contributions and mainly come from the fiber data. A high variation was found in the carbon, aramid, and steel in terms of embodied energy and in the aramid, viscose, and hemp for the GWP. The differences between the first and second cases were insignificant for these samples’ weight, and only noticeable in materials with a remarkably high or low stiffness or strength. For example, the viscose had a higher embodied energy and GWP in the second case. On the contrary, in the same case, the hemp exhibited less ecological burden. The differentiation between Cases 1 and 2 could be higher for other large-scale structures where either stiffness or strength are the dominant design driver.

In comparison to its performance, the aramid required the highest amount of energy to produce and generated the most CO_2_. The carbon showed a substantial contribution to both sustainability markers. The glass and basalt presented the lowest embodied energy, while for GWP, the lowest was flax. The resin contribution was more relevant with samples with a higher FMR _(mass)_ or with fibers with low mass-specific markers, as in the case of the jute. It could also be seen that using bio-based resin helped to reduce the environmental impact, especially in terms of the embodied energy requirements. This reduction was recognizable by comparing C and C*. As the flax (F1 and F1*) samples FMR _(mass)_ were high, this effect could hardly be found in those samples. The flax fibers seemed to be the most suitable material among all the investigated fibers, regarding both its mechanical performance and environmental impact. The deployment of bio-based resins can be justified in this comparison if a lower FMR is present.

## 4. Discussion

### 4.1. Limitations and Advantages of the Assessment

Three different assessments were used in this study: fabrication suitability, structural performance, and sustainability of the selected materials. It should be noted that other crucial aspects not considered, such as long-term behavior and fire resistance will play an important role for real applications.

The structural performance of CFW full-scale structures will highly depend on the consistency of the fabrication; it was essential to reflect the technique uncertainties in the samples to be evaluated. Due to this reason, two types of specimens were designed. One resembled geometry and fiber interaction on a small scale to test the fabrication difficulties of new material systems. The other was a simplified coupon test to compare the structural performance of the materials. For the second sample type, a tensile and compressive axial loading evaluation would have better represented the actual mechanical performance of a CFW structure; however, the four-point bending tests allowed the comparison between the significantly different mechanically performing materials with a common reference parameter, and the structural depth of the sample. Due to the adjustments in sample geometry required by some fiber types, the presented test results of this study may not be directly transferable to other four-point bending results from the literature. Nevertheless, they allowed for obtaining reasonable and comparable results in terms of strength and stiffness, also uncovering possible detriments in the performance resulting from fabrication issues, such as impregnation issues of the fibers or consolidation of the bundles.

The sustainability assessment performed was not meant to give final results but to demonstrate the range in which each material scores. Specific information from each material supplier needs to be collected in order to perform a proper sustainability assessment which was outside the scope of this paper. A complete assessment should also be undertaken for all stages of the material’s life cycle. Further important aspects such as respiratory effects, aquatic contaminations, carcinogens, and land occupation should be included. It is also not entirely realistic to only perform the evaluation at a specimen level; large-scale applications could show different results as the scalability effects have not been investigated. In this study, the results rely on values collected from the literature and are limited to only two markers; however, these results reveal several important aspects. Firstly, the potential of some of the materials in terms of sustainability compared to carbon and glass. Secondly, the variability in the sustainable ranges coming from different suppliers and manufacturers, making crucial the detailed investigation of these aspects prior to the material product selection (e.g., not all the natural fiber products will score as equally sustainable). Thirdly, the lower bound of this range can be frequently associated with developments in the manufacturing processes, namely, towards more sustainable processes. In this context, it is remarkable that the lower range of carbon, especially in terms of embodied energy, matched the upper values of some natural fibers.

In future studies, apart from a testing-supported sustainability assessment and fabrication-related issues, more aspects should also be considered, such as resistance to environmental impacts, aging by ultraviolet irradiation and discoloration, nesting of pests, fungi growth, fire behavior, water absorption, scalability, and design requirements. It should also be taken into account that the absolute numbers of the ecological impact are linked to the available energy mix, which may change with time.

### 4.2. Impact of the Material’s Uncertainties

The novel fabrication technique of CFW produces components with high uncertainties at a macro-structural level coming from fluctuations in the process parameters and the resulting geometry. Substituting the currently used carbon and glass fibers with natural fibers brings additional uncertainties as their properties’ variations translate to an additional inconsistency at the material level. Unpredictable stronger fluctuations translate to higher safety factors and higher material consumption. Since natural fibers generally present a worse mechanical performance compared to the benchmark, it can be expected that significantly increased fiber consumption is needed to achieve the same component structural performance. Higher fiber consumption also translates into a higher resin consumption and this effect could be so dominant that it could affect the design strategies for CFW.

In terms of fabrication, a higher material consumption would manifest into large-scale winding setup designs that tend to use external resin supplies. Fiber feeds would also be needed to reduce the traveling of highly resin-saturated fibers, either by deploying pre-tows or by a direct impregnation system. Another effect would be that winding pins would need to be increased in number or size to accommodate the larger amounts of natural fibers. It should also be noticed that more material for the same component would result in a different architectural appearance, which should be considered for its design.

### 4.3. Scalability of the Equipment and Material System

The system’s scalability in terms of structural capacity should also be evaluated. The increment of material for less performative fibers is not always linear. Structural behaviors such as low stiffness or high ductility can be exponential and critical when scaling up the material system. Even when increasing the material amount correspondingly, it can be expected that not all the materials can be suitable to substitute carbon and glass fibers at any scale or design strategy, especially in long-span applications. This study does not cover the structural system’s scalability effect, a topic that should be further evaluated in future works.

In addition, the adjustments performed in the fabrication domain were designed and proved effective for producing smaller CFW samples; however, some aspects may require further adaptations to transition into a larger architectural scale, such as hairiness, tension limitations, production sessions over several hours, accommodation of the higher material consumption, and different curing methods. Especially for longer winding sessions, fiber fragments generated by natural fibers with pronounced hairiness must be expected to accumulate. Prolonged continuous production may result in a detachment followed by a dislocation of such an accumulation, possibly reducing the diameter of feedthrough in the winding head and thus leading to either reduced resin flow or fibers becoming stuck. This characteristic can be mitigated by reducing the fiber deflection setting, a gentler fiber handling of the dry fibers, providing additional buffer volumes inside the lower impregnation chamber to catch any accumulation, or applying a safety factor to the diameters of the feedthrough. The latter measure is not recommended with natural fibers as the diameters must already be designed relatively large due to the geometrical inconsistencies along the fiber bundle.

It should also be considered that the amount of material processed simultaneously will have to be increased in large-scale productions compared to the material consumption in this study. This adjustment will avoid the proportional increment of the duration of winding sessions, which is limited primarily by the pot life of the resin. To achieve a higher material processing rate, multiple impregnation chambers, one per fiber bundle, should be used in parallel instead of a single chamber processing multiple fiber bundles.

On a larger scale, it would be preferable to use a continuous dryer instead of an oven to set the humidity content of the natural fibers. This adjustment would also avoid any adverse effects on natural fibers resulting from too long or a repeated drying process step. In order to improve fabrication robustness, it would also be advisable to implement an active tension control to the equipment. During curing, a rotation of the samples to achieve a homogeneous resin distribution on hot-curing resin systems with a noticeable drop in viscosity can be needed. This process can be realized on a large scale by an additional rotational axis in the component, which is already usually attached to increase the reach of the robotic system.

### 4.4. Adverse Influences of the Natural Fibers’ Lower Mechanical Properties

Not only the material amount increment and scalability of the system needs to be considered when implementing natural fibers into the CFW process. Further adjustments need to be designed to mitigate the adverse influence of the fibers’ lower mechanical properties.

The limitation in winding tension that applies to fibers with lower absolute strength per fiber bundle, as with natural fibers, is even more crucial with large-scale production because of the longer distances to be traveled between the winding pins, requiring a higher fiber tension. The fiber tension can be calculated according to the catenary equations. This issue could only be solved by geometrical adjustments on the component or by reinforcing the fiber bundle. Geometrical adjustments to reduce the traveling distances could include changes in the winding syntax or a different clustering or position of the winding pins. These adjustments would have a considerable impact on the design strategies. On the other hand, a reinforcement of the fiber bundle could happen by introducing small amounts of a higher dry-fiber-strength fiber material as was already completed in a recent application [24]. To implement the reinforcement of the free-spanning fiber only temporarily would be very impractical to realize. This adjustment could also bring further problems that then would need to be investigated as the two materials would have different mechanical properties that can influence the structural behavior or design of the component.

For materials with lower stiffness, another effect occurs. When spanning the fiber between two points (two winding pins, or winding pin and TCP), the sagging on such materials would be comparatively high. The deflection of the fiber could exceed a tolerable threshold when increasing the distance between both points. This issue could be avoided by applying the same measures as for low-strength materials. Another solution could be implementing a passive tension control at each winding pin. As the pins would move to maintain a constant tension, adapters would be needed to connect to other components later.

The fiber bundle type, which also influences the dry fiber’s mechanical performance, needs to be taken into account. Tapes would have a better structural performance as they are aligned with the expected axial load but would require more adjustments for winding, as previously explained. Yarns are more suitable for winding, but the twist can bring difficulties in the impregnation and the macroscopic bonding between fiber bundles. In this study, the effect of a missing consolidation was not as prominent as it could be in CFW applications, as the samples were produced in a half-open mold. Therefore, a loss in the structural performance of the flax yarn could be expected in large-scale components, which solely rely on consolidation by curvature and pressure applied by the robot pull when winding. With the fabrication adjustments in this study, the flax tape performed better than the flax yarn. This behavior can be justified by the difference in FMR and the better consolidation of the tape. Adjusting the winding process to include a subsequent complete consolidation step entails several economic disadvantages and design limitations to CFW; however, this step could be a valid option to increase the FMR when adjustments to the equipment fail to achieve it.

## 5. Conclusions

This study investigated several aspects: the adjustments needed to the fabrication equipment and processes due to the characteristics of natural fibers, the suitability of selected fiber materials for the CFW technology, and the structural performance and sustainability assessment of the selected alternative fibers supported by structural testing. The aim was to determine if such alternative materials could offer a more sustainable option for CFW than carbon and glass fibers with a petroleum-based epoxy resin. The ranking was normalized in sample performance for the four-point bending test. For reference, two sustainability markers were used: the embodied energy and the global warming potential for raw material production, obtained from the literature.

- It was confirmed in this study that the inconsistencies of natural fibers add to uncertainties in the parameters of CFW components. This variability affects the geomaterial parameters, their inner composition, and as a result, also the structural performance.
- For CFW, the void content should not be neglected, and especially not for natural fibers. The volume-based calculation should rely on the FVR _(area)_, whereas mass-related calculation should be based on the FMR _(mass)_. Converting between FVR and FMR should be avoided, as this would include further uncertainties. Especially for natural fiber composites, methods based on the sample area and mass should be preferred over volume-based methods, such as cuboidal approximation or applying Archimedes’ principle.
- The hairiness, tension limitation, worse impregnation characteristics, and water absorption characteristics entailed by naturals fibers can be mitigated by winding equipment adjustments and process adaptations.
- The inclusion of natural fibers affects large-scale winding setup designs so that they tend to rely on pre-impregnated fibers or a direct impregnation with a resin feed. Adjustments to the size or arrangement of the winding pins or to the winding syntax are needed. Additional elements to help with impregnation and a continuous dryer and tension control, preferably actively controlled, are also needed.
- The resin contribution could be reduced by decreasing the footprint of the resin or by increasing the FMR. Further fabrication adjustments or a subsequent consolidation of the structure can help to reduce this issue.
- In materials with a low FMR, the resin contribution is significant, which can justify using resins with a low ecological impact, such as bio-based epoxies; however, its higher mass-specific price also needs to be considered. In addition, the difference in the obtained FVR between petroleum-based resin and bio-based resin, produced by differences in the fiber’s impregnability by these materials, should also be taken into account.
- The highest lightweight performance is still exhibited by carbon in terms of stiffness, while glass is the better option over carbon for strength because of its economic and ecological benefits. Carbon and glass are not sustainable options if embodied energy and GWP are considered; however, when other architectural or engineering applications such as long-span and low deflection demanding scenarios, the current material choice could still be considered the most viable option.
- Aramid does not offer a higher mass-specific stiffness than carbon but exhibits significantly higher energy consumption and GWP, making it non-preferable for CFW.
- Viscose and steel showed a higher GWP than carbon, although a lower embodied energy; however, viscose especially cannot be competitive with other natural fibers and, therefore, it should not be used for CFW. The recycling potential of steel fibers, which can be fully recycled by melting them down, makes them worthy of further investigation, especially in a scenario where the components are recycled multiple times.
- Based on the approach in this study, flax is found to be the best overall alternative to C/GFRP in CFW as it balances both sustainability markers equally and presents a relatively high mass-specific mechanical performance. Flax also presents an economic advantage over carbon.
- Other materials, such as jute, basalt and hemp, would become suitable options if the resin contribution would be significantly reduced. Specifically, hemp could show a smaller ecological impact since it is a card sliver and not a finished yarn, which removes production steps and, thus, saves resources.
- Under economic aspects, the usage of jute fibers would be interesting, as its price range is low compared to flax (Table 1). The basalt is economically more similar to glass. Here, the price advantage of E-glass over S-glass adds to its ecological benefit.

The research on the alternative materials suitable for CFW application is just at its initial step. This study has uncovered the potentials of some of these materials and the possible future research directions. The investigation of other possible fibers or material combinations should also be considered. The number of sustainability markers could be increased, more products per material type, or a combination of those into a hybrid system could also be incorporated in the research; however, a deeper study on the design influences and scalability of the system is needed.

Replacing virgin with recycled materials could subsequently also be investigated, such as recycled carbon fibers [54]. They could be more competitive as their embodied energy for recycling is at 2.03 MJ/kg [55], significantly lower than the production of new virgin carbon fibers. Despite the very high price, marginal additions of carbon nanotubes or boron fibers could improve the composite properties, especially in compression while cold-curing ceramic matrix systems could increase fire resistance. Alkalic systems could be preferable when using natural fibers and vitrimers could be an approach to improve the recyclability of structures, whereas furan resins could be more sustainable as they do not rely on non-renewable resources.

Ultimately, each material’s mechanical performance and design requirements should be integrated into the current computational design framework for CFW structures [56] as parameters for the design.

## Figures and Tables

**Table 1:** Overview of several reinforcement fibers and thermoset matrix systems collected from the literature, complete ranges and references in Table A1.

Fiber Material	Origin	Density	Tensile Modulus	Tensile Strength	Elong. at Break	Embodied Energy	GWP	Price
–	–	g/cm^3^	GPa	MPa	%	MJ/kg	kg CO_2_-eq./kg	EUR/kg
Abaca	leaf	0.83–1.5	6.2–33.6	400–980	1–12	–	0.1–0.92	€€
Acryl	petrol.	1.18–1.19	2.76–3.3	62–83	3–6.4	175–176.4	5–26	€€€€
Aramid	petrol.	0.98–1.45	48–146	2120–3600	1.5–4.5	600–1651	8.7–104	€€€€€€
Bamboo	grass	0.6–1.5	11–35.91	140–800	1.4–3.7	–	0.0515–3.05	€
Banana	leaf	0.8–1.35	8.5–32	144–800	1.5–30.6	–	0.45–1.04	€
Basalt	mineral	2.15–2.7	42–100	1790–3000	1.12–3.5	6.63–18	0.386–0.986	€€€€
Boron	mineral	2.3–2.61	400–428	3600	1	–	17.78–43.92	€€€€€€€
Carbon	petrol.	1.4–2.2	200–935	1800–6000	0.3–2.1	130–595	12.55–31	€€€€€€
Coconut	leaf	1.15	2.3–18	46.4–500	2.84–5.5	–	0.3286	€
Coir	fruit	1.15–1.46	2.8–6	95–270	15–51.4	10	–	€
Cotton	seed	1.5–1.6	5.5–13	287–800	3–10	5.759–60	0.4341–8	€€€
Flax	bast	1.4–1.5	27–110	343–2000	1.2–3.3	6.5–86	0.4375–0.9	€€
E-glass	mineral	2.5–2.62	70–77	2000–3790	0.5–4.8	8.67–51.3	0.512–4.6	€€€
S-glass	mineral	2.48–2.5	85–103	4480–4890	4.6–5.7	6.013–16	2.452–4.6	€€€€
Hemp	bast	1.4–1.5	3–90	270–1100	1–4	8.89–50	0.531–3	€€
Jute	bast	1.3–1.5	3–55	187–800	0.7–1.8	10–30	0.52–1.12	€
Kenaf	bast	1.22–1.45	4.3–60	223–1191	1.5–2.7	10	5.59	€
Palm	leaf	1.03	2.75	377	13.71	–	–	€
Pineapple	leaf	1.526	60–82	170–1627	2.4–3.2	16.93	4.43	–
Polyamide	petrol.	1.82	0.95	44	18	130–248.4	12.7–37	€€
Polyester	petrol.	1.38	10	1100	22	125–126	2.8–19	€
Ramie	bast	1.0–1.55	24.5–128	220–1000	1.2–4.0	10	1	€€
Silk	animal	1.097–1.34	0.5–10	519.1–1500	18–270	520–580	35	€€€€€
Sisal	leaf	1.3–1.5	9–38	227–955	2–14	10	1	€
Stainless steel	mineral	7.68–8	200	500–1400	5	14–210	2.62–6.8	€€
Viscose	plant	1.5–1.52	11–20	593–830	10.7–13	71–100.8	6.4–15	€€€
Wool	animal	1.3	2–5	100–350	28–61	46.8	7–29.44	€€€
**Resin** **Material**	**Origin**	**Density**	**Tensile** **Modulus**	**Tensile** **Strength**	**Elong. at Break**	**Embodied** **Energy**	**GWP**	**Price**
–	–	g/cm^3^	GPa	MPa	%	MJ/kg	kg CO_2_-eq./kg	EUR/kg
Epoxy	petrol.	1.1–1.4	1.3–6	35–125	1–7.3	76–140.71	5.9–6.75	€€€
Epoxy	bio	1.05–1.159	2–3.3	60–90	2.8–6.1	21.42–43.52	1.42–4.079	€€€€
Phenolics	petrol.	1.2–2.0	0.56–11	20–60	1	130.34	1.34–4.61	–
Polyester	petrol.	1.2–1.5	2–4.5	40–90	2	63–128	3.79–7.6	–
Polyimides	petrol.	1.4	3–4	100–130	5–30	110–340	5.8–19.5	–
Polyurethan	petrol.	1.05	3.1	62.8	9.1	77.83–102.2	3.2–4.56	–

**Table 2:** Overview of the selected fiber materials.

ID	Fiber	Appearance	Avg. Fiber Length **	Tensile Modulus	Tensile Strength	Density	Filaments/Fibers **	Linear Density	Filament/ Fiber Diameter **	Tear Length
–	–	–	mm	GPa	MPa	g/mm^3^	–	tex	µm	km
C/C *	carbon	roving	–	240	4300	1.78	24,000	1600	7	246
G1	E-glass	roving	–	72.5	3450	2.62	1690	2400	24	134
G2	S-glass	roving	–	86.9	4890	2.49	2570	406	9	201
B1	basalt	roving	–	87.5	3000	2.60	4300	2540	17	118
B2	basalt	roving	–	95	1790	2.60	9710	2400	11	70
A	aramid	roving	–	78	3045	1.45	1000	114	12	215
S	steel	roving	–	200	1293	8.00	4500	1920	8	16
V	viscose	roving	–	13.8	710	1.52	20,940	3600	12	48
F1/F1 *	flax	tape	4–900	55.1	875	1.45	12,810	2400	13	62
F2	flax	yarn	4–900	68.3	658	1.45	10,410	2000	13	46
H	hemp	crocheted card sliver	5–140	46.5	690	0.86	2170	17,300	109	82
J	jute	yarn	0.8–120	29.0	493.5	1.46	1050	750	25	34

* The materials marked with an asterisk in the ID were fabricated with the petroleum-based resin instead of the bio-based resin. ** The avg. fiber length was not product specific as it was taken from the literature [23,46,47]. The underlined filament/fiber numbers were calculated by the linear density of the bundle divided by the cross-sectional area and density of the filament/fiber. The underlined filament/fiber diameters were measured by microsections. The underlined tensile modulus and strength were averaged from the literature.

**Table 3:** Overview of the selected resin materials.

Epoxy Resin Type	Density	Stiffness	Strength	Elong. at Break	Viscosity	T_G_	Pot Life	Embodied Energy	GWP
–	g/cm^3^	GPa	MPa	%	mPa*s	°C	min	MJ/kg	kg CO_2_-eq./kg
petrol	1.127	3.15	68	7	1975	106	420	76–139	6.66–6.75
bio	1.075	2.10	80	3	450	115	∞	21.42–43.52	1.42–2.85

**Table 4:** Parameters of the produced four-point bending samples.

ID	Bundles	Height	Width	Length *	Mass	Vol. (geo.)	Vol. (arch.)
–	–	mm	mm	mm	g	cm^3^	cm^3^
C	40	6.51 ± 0.07	15.57 ± 0.80	227 ± 6.05	25.89 ± 1.24	23.02 ± 1.25	20.48 ± 1.26
C*	40	6.61 ± 0.04	17.74 ± 0.87	234 ± 3.77	30.18 ± 1.93	27.48 ± 1.60	23.80 ± 1.16
G1	32	6.47 ± 0.04	15.19 ± 0.65	224 ± 10.26	28.62 ± 1.61	21.97 ± 1.48	19.23 ± 1.13
G2	152	6.53 ± 0.02	13.16 ± 2.01	253 ± 3.86	26.76 ± 4.28	21.58 ± 3.44	17.76 ± 2.83
B1	36	6.62 ± 0.07	16.84 ± 0.92	250 ± 0.99	33.62 ± 1.88	27.88 ± 1.44	22.74 ± 1.15
B2	36	6.62 ± 0.06	16.67 ± 0.64	242 ± 5.46	37.38 ± 3.64	26.64 ± 0.94	23.23 ± 1.36
A	300	6.51 ± 0.05	13.73 ± 0.52	251 ± 1.57	22.22 ± 0.77	22.43 ± 0.85	19.03 ± 0.54
S	46	6.47 ± 0.03	15.58 ± 0.59	250 ±1.97	40.88 ± 2.01	25.19 ± 0.96	21.46 ± 0.78
V	20	6.55 ± 0.06	18.49 ± 1.19	247 ± 4.30	29.61 ± 1.67	29.66 ± 2.14	25.66 ± 1.68
F1	36	6.66 ± 0.12	17.13 ± 0.91	249 ± 0.63	26.62 ± 2.22	28.44 ± 1.78	24.59 ± 1.87
F1*	36	6.66 ± 0.03	17.13 ± 1.22	250 ± 1.10	26.62 ± 1.70	28.44 ± 2.10	24.59 ± 1.74
F2	25	6.69 ± 0.14	18.74 ± 1.18	250 ± 1.73	28.27 ± 2.02	31.36 ± 2.11	25.24 ± 1.49
H	2	6.65 ± 0.06	16.82 ± 0.89	252 ± 2.29	24.99 ± 2.97	27.08 ± 3.24	23.87 ± 2.71
J	40	6.54 ± 0.07	16.25 ± 0.88	250 ± 1.52	23.79 ± 1.39	26.59 ± 1.41	22.94 ± 1.65

* Values given for completeness.

## Data Availability

Data is contained within the manuscript.

## Appendix A

**Table A1:** Table 1 with literature references.

Fiber Material	Density	Tensile Modulus	Tensile Strength	Elong. at Break	Embodied Energy	GWP	Price
–	g/cm^3^	GPa	MPa	%	MJ/kg	kg CO_2_-eq./kg	EUR/kg
Abaca	0.83 [57] 1.5 [30,58]	6.2–20 [30] 12 [58] 12–13.8 [57] 33.6 [59]	400 [58] 400–980 [30] 418–486 [57] 813 [59]	1–10 [30] 3–12 [58]	–	0.1–0.92 [60]	0.31 * [61] 1.377–2.268 * [46]
Acryl	1.18–1.19 [62]	2.76–3.3 [62]	62–83 [62]	3–6.4 [62]	175 [63] 176.4 [64]	5 [63] 26 [64]	2–25 **
Aramid	0.98–1.450 [65] 1.44 [66]	48–146 [66] 55–140 [67] 59–127 [68] 62–131 [69]	2080 [70] 2120 [71] 2400–2700 [66] 2700–2800 [69] 2800 [68] 3600 [65]	1.5–3.3 [66] 2–4.5 [67] 3.7 [68]	600–1350 [72] 1651 [68]	8.7 [73] 13.19 [74] 13.232 [68] 37.5 [72] 90–104 [72]	74.49 * [72]
Bamboo	0.6–1.1 [30] 0.91–1.5 [57]	11–32 [30] 27–35.91 [57] 35.91 [58]	140–800 [30] 500 [58] 503–575 [57]	1.4 [58] 2.5–3.7 [30]	–	0.0515 [75] 3.05 [76]	0.225–0.45 * [46] 0.45 * [61]
Banana	0.8 [59] 1.35 [30]	8.5 [59] 12 [30] 17.85 [58] 27–32 [41]	144–567 [57] 161.8 [59] 500 [30] 600 [58] 700–800 [41]	1.5–9 [30] 2.5–3.7 [41] 3.36 [58] 21.8–30.6 [57]	–	0.45–1.04 [77]	0.8 * [61]
Basalt	2.15 [78] 2.6 [79] 2.7 [80]	42 [78] 60 [80] 90 [45] 100 [81]	1790 [81] 2850 [45] 3000 [82]	1.12 [80] 3.5 [83]	6.63 [78] 18 [79]	0.386 [78] 0.986 [84]	15–20 **
Boron	2.3–2.61 [69] 2.57 [85]	400–428 [69]	3600 [69]	1 [86]	–	17.78–43.92 [87]	180 * [88] 2600 [89]
Carbon	1.4 [23] 1.7–2.1 [69] 1.75–1.8 [66] 1.76 [90] 1.82 [58] 2.2 [91]	200–250 [66] 220–550 [69] 230–240 [23] 240–425 [90] 620–935 [91]	1800–3500 [69] 2600 [91] 3000–5000 [66] 4000 [23] 4100–6000 [90]	0.3–1.4 [69] 1.1–2.1 [90] 1.2–1.4 [66] 1.4–1.8 [23]	130 [61] 183–286 [21,92] 198–595 [93] 338.97 [74] 390–420 [72] 460 [94]	12.55 [74] 20 [95] 24–31 [94]	11.26 * [61] 14.06–15.99 * [96] 24–120 * [72]
Coconut	1.15 [58]	2.3 [59] 2.5 [58] 18 [57]	46.4 [59] 119.8 [57] 500 [58]	2.84 [59] 3.36 [58] 5.5 [57]	–	0.3286 [97]	0.21 [98]
Coir	1.15–1.2 [57] 1.15–1.46 [30] 1.2 [58] 1.25 [99]	2.8–6 [30] 3–6 [92] 4–6 [59] 6 [41,99]	95–230 [30] 106–270 [92] 108–252 [57] 175 [59] 220 [41,99]	15–25 [99] 15–40 [57] 15–51.4 [30] 23.9–51.4 [41] 30 [59]	10 [72]	–	0.18–0.396 * [46] 0.18–0.45 * [61] 0.24–0.48 [92]
Cotton	1.5–1.6 [30] 1.51 [99]	5.5–12.6 [30] 6–13 [47] 12 [41,99]	287–800 [30] 300–600 [47] 400 [41,99]	3–10 [30,99] 7–8 [47]	5.759–32.643 [100] 54 [64] 60 [63]	0.4341 [97] 0.978–2.446 [100] 6 [63] 8 [64]	1.35–3.78 * [61] 1.377–1.98 * [46] 1.61–4.59 [92]
Flax	1.35–3.78 * [61] 1.377–1.98 * [46] 1.61–4.59 [92]	27–80 [57] 27.6 [23] 27.6–103 [30] 30–110 [47] 45–100 [101] 60–80 [41,66,99]	343–2000 [30] 345–1830 [57] 400–2000 [47] 500–1500 [23] 600–1100 [101] 780–1500 [41] 800–1500 [66,99]	1.2–1.6 [66] 1.2–2.4 [41] 1.2–3 [47] 1.2–3.3 [30] 1.5–2.4 [101] 2.7–3.2 [23] [99]	6.5 [21,102] 9.55 [103] 86 [104]	0.4375 [97] 0.9 [105]	0.28–1.377 * [46] 1.89–3.78 * [61] 2.29–11.47 [92]
E-glass	2.5 [23] 2.5–2.59 [46] 2.5–2.6 [30] 2.52–2.6 [66] 2.54 [85] 2.55 [99] 2.56 [101] 2.6 [106] 2.62 [107]	70 [23,106] 70–76 [30] 72 [101] 72–74 [47] 72–77 [66] 72.5 [85] 73 [99]	2000–3400 [101] 2000–3500 [23,30] 2300–2500 [47] 2400 [99,106] 3400–3500 [66] 3450 [85] 3790 [108]	0.5 [23] 1.8–3.2 [101] 1.8–4.8 [30] 3 [99] 3.2–3.5 [47] 3.3–4.8 [66]	8.67 [74] 13–32 [21,92] 17.5 [35] 30 [61] 48.33 [47,99] 51.3 [106]	0.512 [74] 1.8–4.6 [105] 2.04 [47,99] 2.95 [106]	1.08–1.62 * [61] 1.44–2.925 * [46]
S-glass	2.48 [107] 2.49 [85] 2.5 [58]	85 [58] 85–103 [109] 85.6 [85]	4480 [85] 4580 [58] 4630–4890 [109]	4.6 [58] 5.7 [109]	6.013 [110] 16 [35]	2.452 [110] 4.6 [105]	11.9 * [85] 21 [111]
Hemp	1.4–1.5 [30,101] 1.47 [23] 1.48 [66,99]	3–90 [92] 23.5–90 [30] 35 [101] 58–70 [57] 60–70 [47] 70 [23,66,99]	270–900 [30] 389 [101] 550–900 [99] 550–1100 [47] 550–1110 [57] 600–900 [66] 690 [23]	1–3.5 [30] 1.1–1.6 [101] 1.6 [57,66,99] 1.6–1.8 [47] 2–4 [23]	8.89 [106] 18 [64] 30–50 [72]	0.531 [106] 0.5637 [97] 3 [64,72]	0.28–1.486 * [46] 0.57–1.73 [92] 0.9–1.89 * [61]
Jute	1.3 [23] 1.3–1.45 [101] 1.3–1.49 [30] 1.3–1.5 [57] 1.46 [99]	3–55 [92] 10–30 [41,47,99] 10–55 [57] 26.5 [23] 30 [30] 43 [101]	187–773 [92] 320–550 [101] 320–800 [30] 393–773 [23] 393–800 [57] 400–800 [41,47,99]	0.7–1.8 [47] 1–1.8 [30] 1.5–1.8 [23,41,57] 1.7 [101] 1.8 [99]	10 [72] 30 [112]	0.52–1.120 [112] 0.5703 [97]	0.12–0.35 [92] 0.27–0.28 * [46] 0.36–0.5 * [61]
Kenaf	1.22–1.4 [57] 1.4 [30] 1.45 [23,58]	4.3–60 [57] 14.5–53 [30] 22–53 [92] 53 [23]	223–930 [30] 250–1191 [57] 295–930 [92] 930 [23]	1.5–2.7 [30] 1.6 [23,58]	10 [72]	5.59 [100]	0.18 * [72] 0.279–0.585 * [46] 0.53–0.61 [92]
Palm	1.03 [59]	2.75 [59]	377 [59]	13.71 [59]	–	–	0.07 [98]
Pineapple	1.526 [59]	60–82 [59] 82 [41]	170–1627 [59] 180 [41]	2.4 [59] 3.2 [41]	16.93 [113]	4.43 [114]	–
Polyamide	1.82 [115]	0.95 [116]	44 [115]	18 [116]	130 [63] 248.4 [64]	12.7 [117] 37 [64]	1.01–1.55 [118]
Polyester	1.38 [66]	10 [66]	1100 [66]	22 [66]	125 [63] 126 [64]	2.8 [63] 12.7 [119] 19 [64]	0.42–0.48 [118]
Ramie	1.0–1.55 [30] 1.4–1.5 [101] 1.5 [58,99]	24.5–128 [30,57] 44 [41,99] 44–128 [58] 61.4–128 [23] 128 [101]	220–938 [58] 400–938 [23] 400–1000 [30,57] 500 [99] 500–870 [41] 500–1000 [101]	1.2 [41] 1.2–4.0 [57,101] 2 [99] 2–3.8 [58] 3.6–3.8 [23] [30]	10 [72]	1 [72]	1.377–2.17 * [46] 1.8 * [61]
Silk	1.097 [120] 1.34 [121]	0.5–1.1 [116] 6.2 [122] 10 [121]	519.1 [122] 1500 [121]	18–270 [116]	520–580 [72]	35 [72]	30 * [72]
Sisal	1.3–1.5 [59] 1.33 [99] 1.33–1.5 [30] 1.45 [58] 1.5 [23]	9–28 [57] 9–38 [30] 9.4–22 [23,58] 17–22 [41] 28 [59] 38 [99]	227–885 [57] 363–700 [30] 507–955 [59] 511–635 [23] 530–630 [41] 530–640 [58] 600–700 [99]	2.0–2.5 [23] 2–2.9 [59] 2–3 [99] 2.0–7.0 [30] 2–14 [57] 3–7 [58] 3.64–5.12 [41]	10 [72]	1 [72]	0.315–0.585 * [46] 0.54–0.63 * [61]
Stainless steel	7.68 [79] 7.98 [123] 8 [124]	200 [124]	500–700 [124] 1400 [123]	5 [123]	14 [79] 30–60 [21] 30.37 [99] 32.1 [125] 49–80 [126] 110–210 [56]	2.62 [99] 4.9–6.8 [126] 5.18414 [127]	0.8–2.7 **
Viscose	1.5 [128,129] 1.52 [130]	11 [57] 14.3–16.5 [128] 20 [129]	593 [57] 682–778 [128] 830 [129]	10.7–12.7 [128] 11 [57] 13 [129]	71 [63] 100.8 [64]	6.4 [131] 14 [132] 15 [64]	2–12 **
Wool	1.3 [133]	2–5 [34]	100–350 [34]	28–61 [34]	46.8 [64]	7 [64] 29.44 [134]	2.42 * [72]
**Resin** **Material**	**Density**	**Tensile** **Modulus**	**Tensile** **Strength**	**Elong. at Break**	**Embodied** **Energy**	**GWP**	**Price**
–	g/cm^3^	GPa	MPa	%	MJ/kg	kg CO_2_-eq./kg	EUR/kg
Epoxy	1.1–1.4 [30,69] 1.123 [106] 1.2 [66]	1.3–3.5 [69] 3–6 [30,66] 3.1–3.2 [101] 3.5 [106] 3.9 [135]	35–100 [30,69] 47.7 [135] 60–125 [66] 69 [106] 76 [101]	1–6 [30] 1.5 [135] 7.3 [101]	76–80 [21,92] 122.4 [97] 135.04 [74] 139 [136] 140.71 [47]	5.9 [47] 6.663 [97,106] 6.7 [136] 6.75 [74]	7.66 **
Epoxy	1.05–1.1 [137] 1.12 [106] 1.159 [138]	2–2.2 [137] 3–3.3 [138] 3.074 [106]	60.0 [106] 70–90 [137] 71–81 [138]	2.8–6.1 [138] 3 [137]	21.42–43.52 **	1.42–2.85 ** 4.079 [97,106]	15–20 **
Phenolics	1.2–1.3 [69] 1.3–2.0 [66]	0.56–2.5 [66] 5–11 [69]	20–60 [66] 50–60 [69]	1 [139]	130.34 [74]	1.34 [140] 4.61 [74]	–
Polyester	1.2 [66,69] 1.2–1.5 [30] 1.35 [141]	2–3 [69] 2–4.5 [30] 3–4.2 [66] 3.23 [141]	40–70 [66] 40–90 [30] 50–60 [69] 85 [141]	2 [30]	63–78 [21,92,102] 128 [136]	3.79 [74] 7.6 [136]	–
Polyimides	1.4 [69]	3–4 [69]	100–130 [69]	5–30 [142]	110–340 [143]	5.8–19.5 [143]	–
Polyurethan	1.05 [144]	3.1 [135]	62.8 [135]	9.1 [135]	77.83–102.2 [74]	3.2–4.56 [74]	–

* Prices were converted into EUR (1.00 USD = 0.90 EUR, 1.00 GBP = 1.21 EUR). ** Values are based on several individual products.
